# Metagenomic insights into the taxonomy, function, and dysbiosis of prokaryotic communities in octocorals

**DOI:** 10.1186/s40168-021-01031-y

**Published:** 2021-03-25

**Authors:** T. Keller-Costa, A. Lago-Lestón, J. P. Saraiva, R. Toscan, S. G. Silva, J. Gonçalves, C. J. Cox, N. Kyrpides, U. Nunes da Rocha, R. Costa

**Affiliations:** 1 Instituto de Bioengenharia e Biociências (iBB), Instituto Superior Técnico (IST), Universidade de Lisboa, Av. Rovisco Pais 1, 1049-001 Lisbon, Portugal; 2 División de Biología Experimental y Aplicada (DBEA), Centro de Investigación Científica y de Educación Superior de Ensenada (CICESE), Carr. Ensenada-Tijuana 3918, Zona Playitas, C.P 22860 Ensenada, Baja California Mexico; 3 Helmholtz Centre for Environmental Research (UFZ), Leipzig, 04318 Germany; 4 Centro de Ciências do Mar (CCMAR), Universidade do Algarve, 8005-139 Faro, Portugal; 5 Department of Energy, Joint Genome Institute, Berkeley, CA 94720 USA

**Keywords:** Host-microbe interactions, Symbiosis, Holobiont, Gorgonians, *Eunicella*, *Leptogorgia*, Secondary metabolism, Necrosis

## Abstract

**Background:**

In octocorals (Cnidaria Octocorallia), the functional relationship between host health and its symbiotic consortium has yet to be determined. Here, we employed comparative metagenomics to uncover the distinct functional and phylogenetic features of the microbiomes of healthy *Eunicella gazella*, *Eunicella verrucosa*, and *Leptogorgia sarmentosa* tissues, in contrast with the microbiomes found in seawater and sediments. We further explored how the octocoral microbiome shifts to a pathobiome state in *E. gazella*.

**Results:**

Multivariate analyses based on 16S rRNA genes, Clusters of Orthologous Groups of proteins (COGs), Protein families (Pfams), and secondary metabolite-biosynthetic gene clusters annotated from 20 Illumina-sequenced metagenomes each revealed separate clustering of the prokaryotic communities of healthy tissue samples of the three octocoral species from those of necrotic *E. gazella* tissue and surrounding environments. While the healthy octocoral microbiome was distinguished by so-far uncultivated *Endozoicomonadaceae*, *Oceanospirillales*, and *Alteromonadales* phylotypes in all host species, a pronounced increase of *Flavobacteriaceae* and *Alphaproteobacteria*, originating from seawater, was observed in necrotic *E. gazella* tissue. Increased abundances of eukaryotic-like proteins, exonucleases, restriction endonucleases, CRISPR/Cas proteins, and genes encoding for heat-shock proteins, inorganic ion transport, and iron storage distinguished the prokaryotic communities of healthy octocoral tissue regardless of the host species. An increase of arginase and nitric oxide reductase genes, observed in necrotic *E. gazella* tissues, suggests the existence of a mechanism for suppression of nitrite oxide production by which octocoral pathogens may overcome the host’s immune system.

**Conclusions:**

This is the first study to employ primer-less, shotgun metagenome sequencing to unveil the taxonomic, functional, and secondary metabolism features of prokaryotic communities in octocorals. Our analyses reveal that the octocoral microbiome is distinct from those of the environmental surroundings, is host genus (but not species) specific, and undergoes large, complex structural changes in the transition to the dysbiotic state. Host-symbiont recognition, abiotic-stress response, micronutrient acquisition, and an antiviral defense arsenal comprising multiple restriction endonucleases, CRISPR/Cas systems, and phage lysogenization regulators are signatures of prokaryotic communities in octocorals. We argue that these features collectively contribute to the stabilization of symbiosis in the octocoral holobiont and constitute beneficial traits that can guide future studies on coral reef conservation and microbiome therapy.

Video Abstract

**Supplementary Information:**

The online version contains supplementary material available at 10.1186/s40168-021-01031-y.

## Background

Communities of beneficial, commensal, and opportunistic microbes are an integral part of most animals, and together, they may form an ecological unit, called the holobiont [1]. Corals are no exception [2], and coral-microbiome interactions are assumed to play a key role in host’s resilience in a climate change scenario. However, our ability to benefit from such interactions in conservation practices is severely limited due to our lack of understanding of the microbial functions that are indispensable for holobiont health and functioning.

Octocorals (Cnidaria, Anthozoa, Octocorallia), characterized by the eight-fold symmetry of their polyps, comprise a lineage of metazoans that diverged from scleractinian corals (Hexacorallia) more than 540 million years ago [3]. Over 3500 octocoral species have been described to date [4]. Octocorals are distributed worldwide [5] and are model organisms in the study of the evolution of the metazoan immune and hormonal system. In temperate marine biomes, octocorals can make up to 95% of the total biomass, dictating carbon flux dynamics, increasing benthic biodiversity, and functioning as true ecosystem engineers [6, 7].

As with other scleractinian corals, octocorals live in symbiosis with complex microbial communities. However, current knowledge of the structure and function of the octocoral-associated microbiome is still relatively scant. Many temperate octocoral species are azooxanthellate (in the Mediterranean Sea, more than 95%), meaning they lack the photosynthetic *Symbiodiniaceae* symbionts [8]. Instead, microbial associations in these octocorals are presumed to be dominated by chemotrophic prokaryotes [8]. Most octocoral microbiome studies have been restricted to 16S rRNA gene-based taxonomic profiling, yet they have revealed that octocoral bacterial communities are diverse, distinct from seawater, and often host species specific [8–14]. Their bacterial assemblages are commonly dominated by *Proteobacteria*, particularly *Gammaproteobacteria* belonging to the *Oceanospirillales* genus *Endozoicomonas*, which can make up to 96% of an octocoral’s prokaryotic community and are considered core symbionts [8–12, 15–18]. Some *Endozoicomonas* phylotypes are capable of dimethylsulfoniopropionate (DMSP) metabolization and are thought to play a role in sulfur cycling within their host [17, 19]. The maintenance of a functional microbiome seems essential to coral holobiont fitness [20], and bacterial symbionts of corals have been implicated in several important functions such as carbon acquisition [17, 21, 22], nitrogen fixation [8, 17, 23], sulfur cycling [8, 17, 19, 24], phosphorous supply [17], and biosynthesis of antimicrobial compounds [25, 26]. Indeed, octocorals and their microbial associates are prolific producers of bioactive compounds with ca. 200 novel structures described yearly [27] and hence present an enormous potential for the blue economy sector [28].

Abiotic and biotic stressors such as infectious diseases and climate change have led to significant mortalities in Mediterranean and North-East Atlantic octocoral populations during the past two decades [29–33]. Such mortality events can alter critical ecosystem processes and result in biodiversity loss in the benthos of temperate zones [30]. Despite their devastating impacts, the community shifts underpinning microbiome dysbiosis, tissue necrosis, and ultimately mortality in octocorals remain largely unknown. A 16S rRNA gene-based pyrosequencing study [34] and a few, bacterial cultivation-based studies have pointed towards infections with *Vibrio* pathogens as disease-causing agents [31, 32]; however, holistic experimental and/or omics-based investigations are still lacking. Such studies are necessary to reveal the functional features of the microbiomes of healthy octocorals and the environmental and biotic triggers of disease, and to develop effective mitigation strategies in the long term.

In this study, we aimed to, firstly, determine whether the functional and taxonomic structure of prokaryotic communities inhabiting temperate octocorals are (i) host species specific and (ii) distinct from those of the environmental vicinities and, secondly, to reveal functions that could serve as indicators of octocoral holobiont health. To this end, we employed shotgun metagenome sequencing of the microbiomes of healthy tissue samples from three octocoral species, *Eunicella gazella* (*N* = 3), *E. verrucosa* (*N* = 4), and *Leptogorgia sarmentosa* (*N* = 3), in addition to necrotic *E. gazella* tissue (*N*=3) (Additional file 1: Figure S1), and their surrounding seawater (*N* = 4) and sediment (*N* = 3). To reveal shifts in prokaryotic community structures between the different octocoral species, their health states, and surrounding environments, we firstly performed taxonomic 16S rRNA gene-based profiling of all 20 metagenomes. Subsequently, we used Clusters of Orthologous Groups of proteins (COGs) and Protein families (Pfam)-based annotation to reveal differentially abundant functions across the prokaryotic communities in octocorals (health and necrotic tissues) and seawater. Because octocorals are well-known to be prolific sources of natural products with a range of bioactivities, these functional assessments were complemented with an examination of the abundance, diversity, and phylogenetic relationships of secondary metabolite biosynthetic gene clusters (BGCs) detected in the samples, thereby illuminating shifts in secondary metabolism and the potential for natural product biosynthesis across octocoral and seawater microbiomes.

## Results

### Overview of the microbial metagenome dataset

**Fig. 1:**
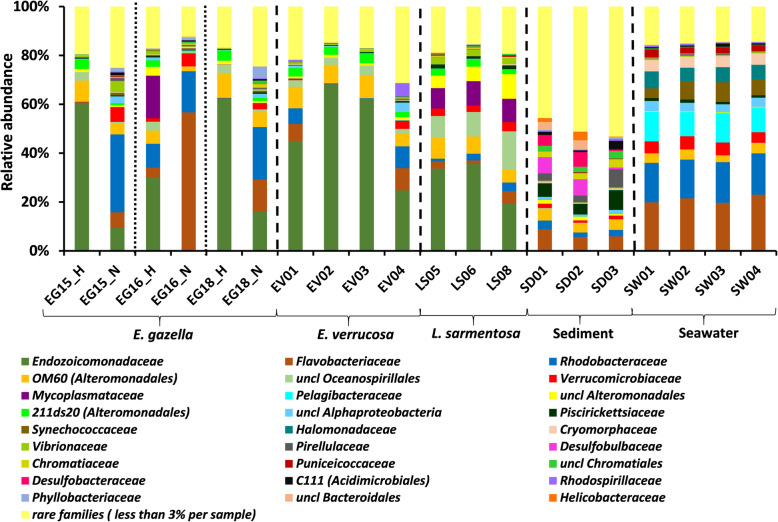
Prokaryotic community composition of octocoral samples, seawater, and sediment. Family-level community profiles of healthy (EG_H) and necrotic (EG_N) *Eunicella gazella* tissue, healthy *Eunicella verrucosa* (EV01-EV04), and *Leptogorgia sarmentosa* (LS06-LS08) specimens as well as seawater (SW01-SW04) and sediment samples (SD01-SD03). Relative abundances are displayed for taxa representing more than 3% of the total dataset reads. Taxa with abundances below 3% across the data are collectively labeled as “rare families”

**Fig. 2:**
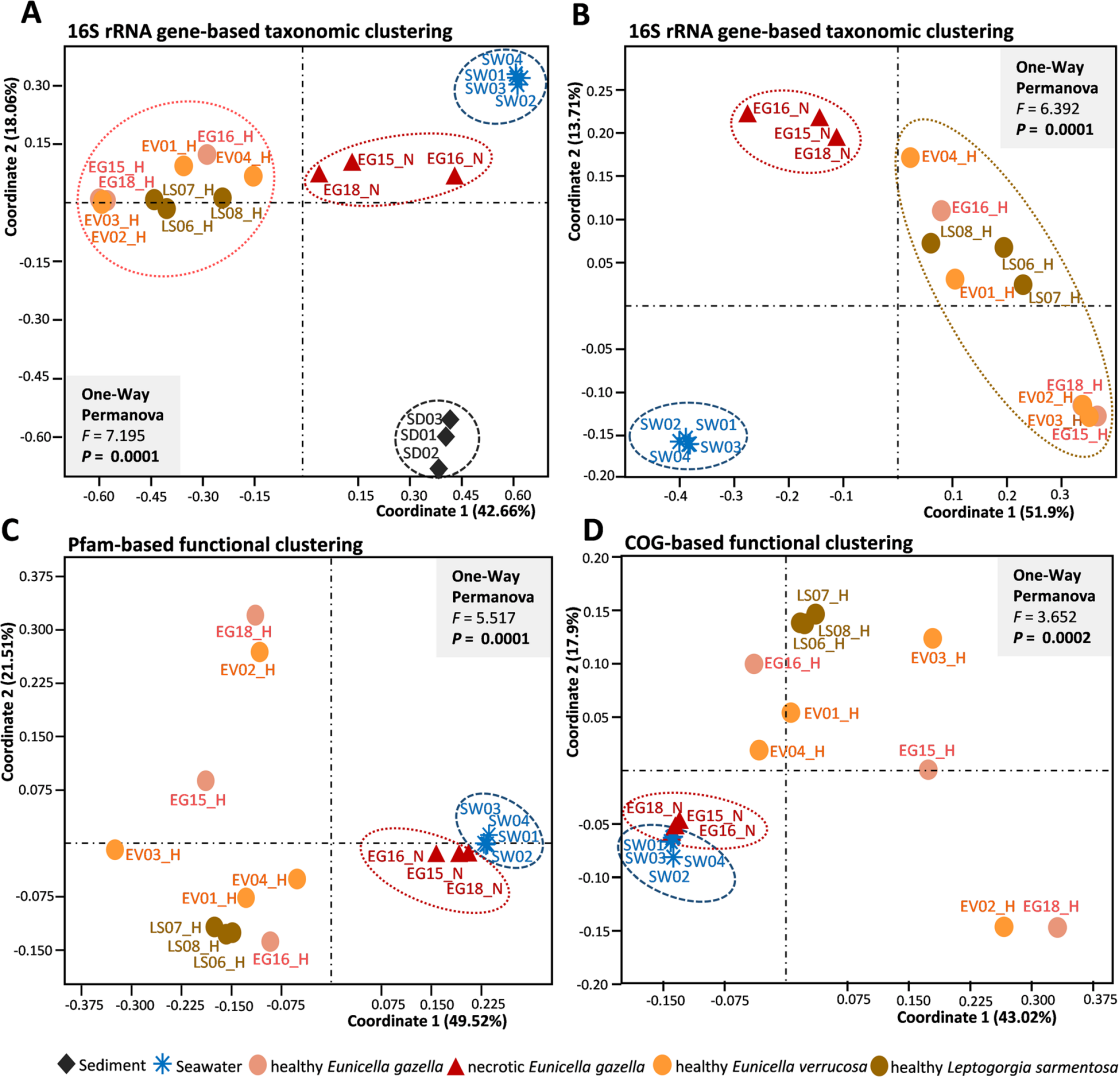
Multivariate analysis of the prokaryotic community profiles. Ordinations are shown at the taxonomic (phylotype, OTU) (**a** and **b)** and functional (Pfam and COG) (**c** and **d)** levels. In **a**, sediment samples were included in the ordination analysis while **b** shows the same ordination without sediment data. Principal coordinates analyses (PCoA) were performed using the Bray-Curtis similarity matrix calculated from Hellinger-transformed abundance data. All ordinations are shown in Eigenvalue scale. Healthy octocoral samples are represented by colored circles (salmon — healthy *Eunicella gazella* (EG15_H - EG18H); orange — healthy *Eunicella verrucosa* (EV01 - EV04); olive — healthy *Leptogorgia sarmentosa* (LS06 - LS08)), necrotic *E. gazella* (EG15_N - EG18_N) by red triangles, sediment (SD01 - SD03) by black diamonds, and seawater (SW01 - SW04) by blue asterisks. Discrete grouping of the sample categories was statistically supported by one-way PERMANOVA tests with each 999 permutations

About 20 million paired end reads of 101 nucleotides in length were generated per sample. In total, 46.12 Gb of sequencing information was obtained across the 20 metagenome samples analyzed in this study (Additional file 2: Table S1). A total of 291,597,021 high-quality unassembled reads (averaging 14,579,851 reads per sample) were scanned by the MGnify v.2.0 metagenomics pipeline from the European Bioinformatics Institute (EMBL-EBI) [35] for the detection of both protein-coding sequences (CDS) and rRNA genes (Additional file 2: Table S1). The latter dataset was used in this study to delineate and compare the prokaryotic, 16S rRNA gene-based taxonomic profiles of all samples (Figs. 1 and 2a, b). Functional analyses were performed on assembled metagenomes (Additional file 2: Table S2), using the Integrated Microbial Genomes & Microbiomes (IMG/M) annotation pipeline from the Joint Genome Institute (DOE-JGI—see “Methods” for details). For more information on the features of the 16S rRNA gene dataset used in taxonomic profiling and of the metagenome assemblies subjected to functional analysis, see Additional file 1: extended results.

### Distinct alpha-diversity measures revealed for octocoral-associated and environmental prokaryotic communities

Alpha-diversity analyses encompassed 93,589 high-quality reads classified into 1041 prokaryotic OTUs (12 archaeal and 1029 bacterial OTUs defined at 97% 16S rRNA gene similarity) obtained from the 20 unassembled metagenome samples (Additional file 2: Tables S3 and S4). Our primer-less, shotgun metagenomic approach showed reduced OTU richness in healthy octocoral specimens compared with surrounding environments. OTU richness and (Shannon’s) diversity measures were significantly higher (*t*-test, *P* = 0.001 and *P* = 0.0173) in necrotic than in healthy *E. gazella* tissue (see Additional file 1: extended results for details).

### Octocoral-associated and environmental prokaryotic communities are divergent at high taxonomic ranks

Congruent with the notion of highly divergent prokaryotic communities being found in distinct habitats, differences in taxonomic composition of the microbiomes of necrotic and healthy octocoral tissue, sediments, and seawater (hereafter termed “sample categories”) were observed even at high taxonomic ranks (e.g., phylum and class levels). *Proteobacteria* was the most dominant phylum across the entire dataset. However, the relative abundance of *Proteobacteria* varied markedly between sample categories, ranging from highly dominant 80 to 98% proportions in healthy octocoral tissues (depending on the species) while dropping to 62% in necrotic *E. gazella* tissue and to 54% and 53% in sediment and seawater, respectively. Besides *Proteobacteria*, *Bacteroidetes* (28.1%), *Verrucomicrobia* (7.8%), and *Cyanobacteria* (7.1%) were the most abundant phyla in the highly consistent taxonomic profiles of seawater, while *Bacteroidetes* (16.9%), *Planctomycetes* (6.7%), and *Actinobacteria* (5.0%) ranked as the next most abundant phyla in sediments. The highest number of prokaryotic phyla (Additional file 2: Table S3), classes (Additional file 1: Figure S2), families (Fig. 1), and OTUs (see above) among all sample categories was found in sediments, highlighting the complex nature of these prokaryotic communities. Among healthy octocoral tissues, the class *Mollicutes* in the *Tenericutes* phylum possessed a consistent pattern of association with *L. sarmentosa*, but not with the two *Eunicella* species examined here (Additional file 1: Figure S2). Notably, *Poribacteria* that are usually regarded as “sponge-specific” were the only phylum that was present in the healthy tissue of all three octocoral species (despite its low relative abundance), but absent in necrotic *E. gazella*, sediment, and seawater. The class *Gammaproteobacteria* within the *Proteobacteria* phylum dominated the prokaryotic communities in healthy tissues of all host species, while a drastic shift towards dominance of the classes *Alphaproteobacteria* (*Proteobacteria*) and *Flavobacteriia* (*Bacteroidetes*) was observed in necrotic *E. gazella* tissues (see Additional file 1: extended results for more details on class abundance distributions across sample categories).

Healthy octocoral samples (all three species together) hosted a total of 220 prokaryotic families (across 37,174 analyzed reads), whereby *E. gazella* hosted 127 (20,420 reads), *E. verrucosa* 174 (13,435 reads), and *L. sarmentosa* 123 (3319 reads) families. In comparison, 252, 263, and 396 families were detected in seawater (26,039 reads), necrotic *E. gazella* tissue (19,927 reads), and sediments (10,449 reads), respectively. This reinforces the notion that host-selection processes occur at taxonomic ranks higher than phylotype (i.e., “OTUs”) level and are a major driver of divergent prokaryotic community structures in octocoral-associated and free-living settings (Fig. 1). *Rhodobacteraceae*, *Flavobacteriaceae*, and *Verrucomicrobiaceae* were dominant members of the microbiomes of seawater and necrotic *E. gazella* tissue, while *Endozoicomonadaceae* and uncultivated families OM60 and 211ds20 (both in the order *Alteromonadales*) characterized the healthy octocoral tissue across all examined species (Fig. 1). Several bacterial families showed a more consistent association and higher relative abundances in *L. sarmentosa* than in the *Eunicella* species, including *Mycoplasmataceae* (*Mollicutes*, *Tenericutes*) and *Vibrionaceae*, and so-far unclassified families in the *Alteromonadales* and *Oceanospirillales* orders (Fig. 1).

At the finest level of taxonomic resolution used in this study (i.e., OTUs), ordination analysis revealed that the prokaryotic communities of healthy octocoral tissue, necrotic *E. gazella* tissue, sediments, and seawater are significantly different from one another (Fig. 2a, b). While the prokaryotic communities of healthy octocorals were distinguished from the other sample categories along the horizontal axis of the ordination diagram, sediment samples clustered far apart from all other sample categories along the vertical axis (Fig. 2a). However, when sediment samples were omitted from the analyses, we could observe the clustering of seawater samples away from octocoral samples (Fig. 2b). This is congruent with the observation that families such as *Synechococcaceae*, *Pelagibacteraceae*, *Halomonadaceae*, and *Cryomorphaceae* (Fig. 1) were characteristic of seawater but were found to be absent or in very low abundance in octocoral samples. Among the healthy tissue samples of all octocoral species, those from *L. sarmentosa* formed a more cohesive cluster in the ordination diagram than the samples representing both *Eunicella* species where higher dispersion in community profiles was observed (see below).

From a total of 1041 OTUs detected across the data, 89 OTUs were common to healthy octocoral tissue, seawater, and sediments, of which only 25 OTUs were present in every single metagenome sample from these groups (Additional file 1: Figure S3a, b). Nine OTUs were shared by healthy tissue samples of all octocoral species while not being present in any of the sediment or seawater samples. Of these, three OTUs stood out, being present in all 10 healthy tissue samples: uncl. *Endozoicomonadaceae* (OTU_866), *Shewanella* (OTU_805), and *Pseudoalteromonas* (OTU_943). Higher proportions of “specific” OTUs were identified in seawater (28%) and sediment (44%) samples than in the three octocoral species, which harbored between 5% (*L. sarmentosa*) and 13% (*E. verrucosa*) unique, species-specific OTUs (Additional file 1: Figure S3a).

### Necrotic octocoral tissue is heavily colonized by typical seawater bacteria

**Fig. 3:**
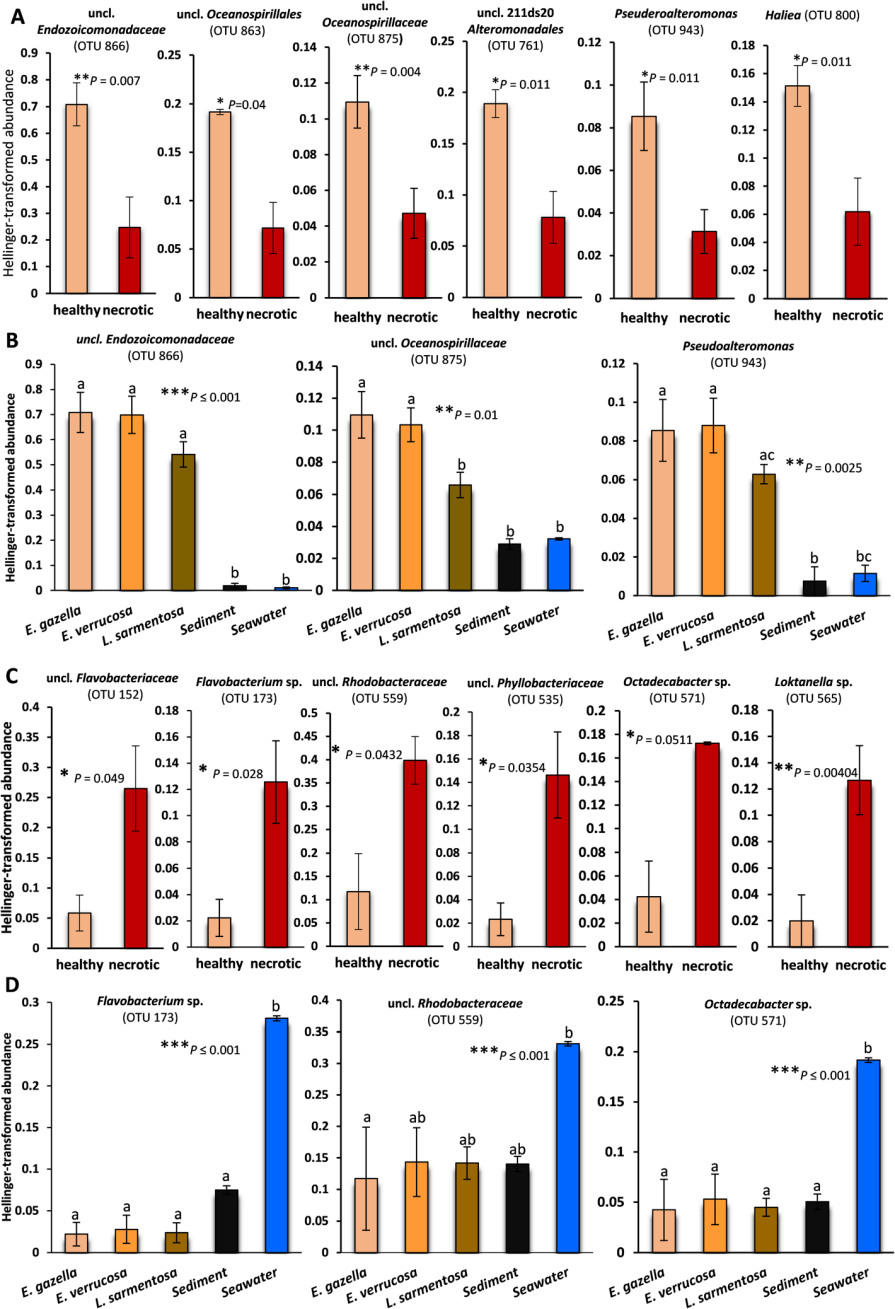
Bacterial phylotypes which are significantly enriched in healthy (**a**) or necrotic (**c**) *Eunicella gazella* tissue. For comparison, the respective abundances of some of these phylotypes (OTUs) in the other octocoral species, sediment, and seawater are shown in **b** and **d**. Bars represent average proportions (%) ± standard errors. Paired *t*-tests (**a** and **c**) or one-way ANOVAs (**b** and **d**) followed by Bonferroni tests were used to check for significant differences between sample groups. Statistical significance was established at *P*-values ≤ 0.05. Letters or asterisks above error bars indicate significant differences (*P ≤ 0.05; **P ≤ 0.01; ***P ≤ 0.001). Respective *P-*values are presented in the graphs. All phylotypes shown here are among the top ten phylotypes respectively enriched in healthy or in necrotic *E. gazella* tissue (Additional file 2: Table S4b,c) that contributed most to the dissimilarities between these microbiomes, as revealed by SIMPER and “Indicspecies” analyses

The 10 most enriched OTUs in healthy octocoral tissue—identified by similarity percentage (SIMPER) analyses—comprised currently unclassified and uncultured *Alteromonadales* and *Oceanospirillales/Endozoicomonadaceae* phylotypes, as well as *Pseudoalteromonas*, *Haliea*, and *Mycoplasma* sp. (*Mollicutes*, *Tenericutes*). While most of these phylotypes were usually abundant in all octocoral species investigated (e.g., OTUs 866, 875, and 943, representing *Endozoicomonadaceae*, *Oceanospirillaceae*, and *Pseudoalteromonas* spp., respectively—Fig. 3), *Mycoplasma* sp. (OTU 1008, Additional file 2: Table S4a) was consistently dominant only in *L. sarmentosa*. Nevertheless, the relative abundances of these taxa were significantly reduced in necrotic tissues and in the surrounding sediment and seawater (Fig. 3a, b; Additional file 2: Table S4b). Thus, they could be considered signatures of the healthy, temperate gorgonian microbiome. This interpretation was further supported by an “Indicspecies” [36, 37] analysis, here used to assess the association between OTUs and health states in *E. gazella* (Additional file 2: Table S4c). “Indicspecies” returned 17 OTUs associated with healthy *E. gazella* tissue and 93 OTUs associated with necrotic *E. gazella* tissue. From those 17 OTUs, nine were also among the top 10 most differentiating phylotypes identified in our SIMPER test. Sixteen of the 17 phylotypes found to be associated with healthy tissue by the “indicspecies” analysis belonged to *Gammaproteobacteria*, most often in the orders *Oceanospirillales* and *Alteromonadales*, demonstrating the high level of congruency between the results of different statistical tests conducted in this study.

In contrast, various cultivable and so-far uncultivable *Flavobacteriaceae*, *Rhodobacteraceae*, and *Verrucomicrobiales* phylotypes, including transient taxa such as *Tenacibaculum*, *Flavobacterium*, *Aquimarina*, *Octadecabacter*, and *Loktanella*, which have been previously cultivated from marine hosts [9, 38–41], were enriched in necrotic octocoral tissue. While their abundance was generally low in healthy tissue of all three octocoral species, it sharply increased in seawater (Fig. 3c, d; Additional file 2: Table S4b). This points to a pronounced colonization of the necrotic octocoral tissue by seawater taxa. Further evidence for opportunistic colonizers from surrounding environments taking control of necrotic tissue communities was provided by Venn diagram analysis. This showed that necrotic *E. gazella* tissue shared over 40% of its OTUs exclusively with seawater and/or sediment, while only 5% were shared exclusively with healthy *E. gazella* tissue (Additional file 1: Figure S3c, d).

No significant change in abundance of *Vibrio* spp. (five *Vibrio* spp. OTUs were present in the dataset) was observed between healthy and necrotic *E. gazella* tissue (paired *t*-test, *P* = 0.703). In addition, no *Vibrio* OTU was among those 93 OTUs identified by the “Indicspecies” analysis to be associated with the necrotic state. However, healthy *L. sarmentosa* samples had a higher abundance of *Vibrio* spp. phylotypes (ANOVA, *P* = 0.0035) than healthy *E. gazella* and *E. verrucosa*, sediment, and seawater. The latter four sample categories shared similar *Vibrio* spp. abundances.

### Functional profiling of prokaryotic communities in octocorals and seawater

For functional comparisons, we focused on prokaryote-enriched metagenome assemblies, after eukaryotic contigs had been filtered out (Additional file 2: Table S5). A total of 7742 Pfam and 4570 COG entries were identified. The number of Pfams identified per sample group ranged from 4286 in seawater to 4887 in healthy *L. sarmentosa*, while the number of COGs ranged from 3473 in healthy *E. gazella* to 3914 in seawater. The full Pfam and COG entry lists for all assembled metagenome samples, and corresponding ANOVA results for each entry, are provided as supplementary information (Additional file 2: Table S6 and S7).

### Ordination analysis of functional profiles

Both Pfam- and COG-based multivariate analyses (Fig. 2c, d) showed significant differences between sample categories (one-way PERMANOVA, *P* < 0.001). Pairwise comparisons revealed that seawater functional profiles differed from those of each octocoral species in both Pfam- and COG-based annotations. Moreover, in the Pfam-based analysis, we found differences between some, but not all, octocoral sample groups, with *E. verrucosa* profiles being different from *L. sarmentosa* and necrotic *E. gazella*. In both, COG and Pfam-based functional analyses, necrotic *E. gazella* samples clustered more closely to seawater, suggesting a higher functional overlap between these two prokaryotic communities.

Healthy octocoral samples (all three species together) showed a more dispersed pattern of distribution in the PCoA plots, pointing towards a higher functional variation and plasticity within the healthy octocoral tissue. In this respect, functional profiles corroborated OTU-level taxonomic profiles in which healthy octocoral samples also showed, altogether, a large within-group variation and some degree of overlap between samples from different host species. However, we note that *L. sarmentosa* samples formed more concise clusters in both taxonomic- and functional-based ordinations (Fig. 2), hinting at a more selective structuring of prokaryotic communities by this species than by *Eunicella* spp. Tests on the multivariate homogeneity of group dispersion (variances) corroborated this observation, showing overall significant differences between sample groups in all taxonomic and functional analyses (16S-OTU (*p* = 0.018), COG (*p* = 0.0065), Pfam (*p* = 0.0074)). Pairwise comparisons revealed that the dispersion among the OTU profiles of the coral sample groups was similar (homogenous) while the dispersion of seawater profiles was significantly smaller than that of healthy and necrotic *E. gazella* samples. Dispersion among COG profiles from seawater samples was significantly smaller than those of healthy coral samples but similar to those of necrotic coral samples. Overall, the highest levels of dispersion were observed in the microbiomes of healthy tissue samples from both *Eunicella* species, followed by healthy *L. sarmentosa* and then necrotic *E. gazella*, while the dispersion in seawater was smallest.

### Functional signatures of prokaryotic communities in healthy octocorals

**Fig. 4:**
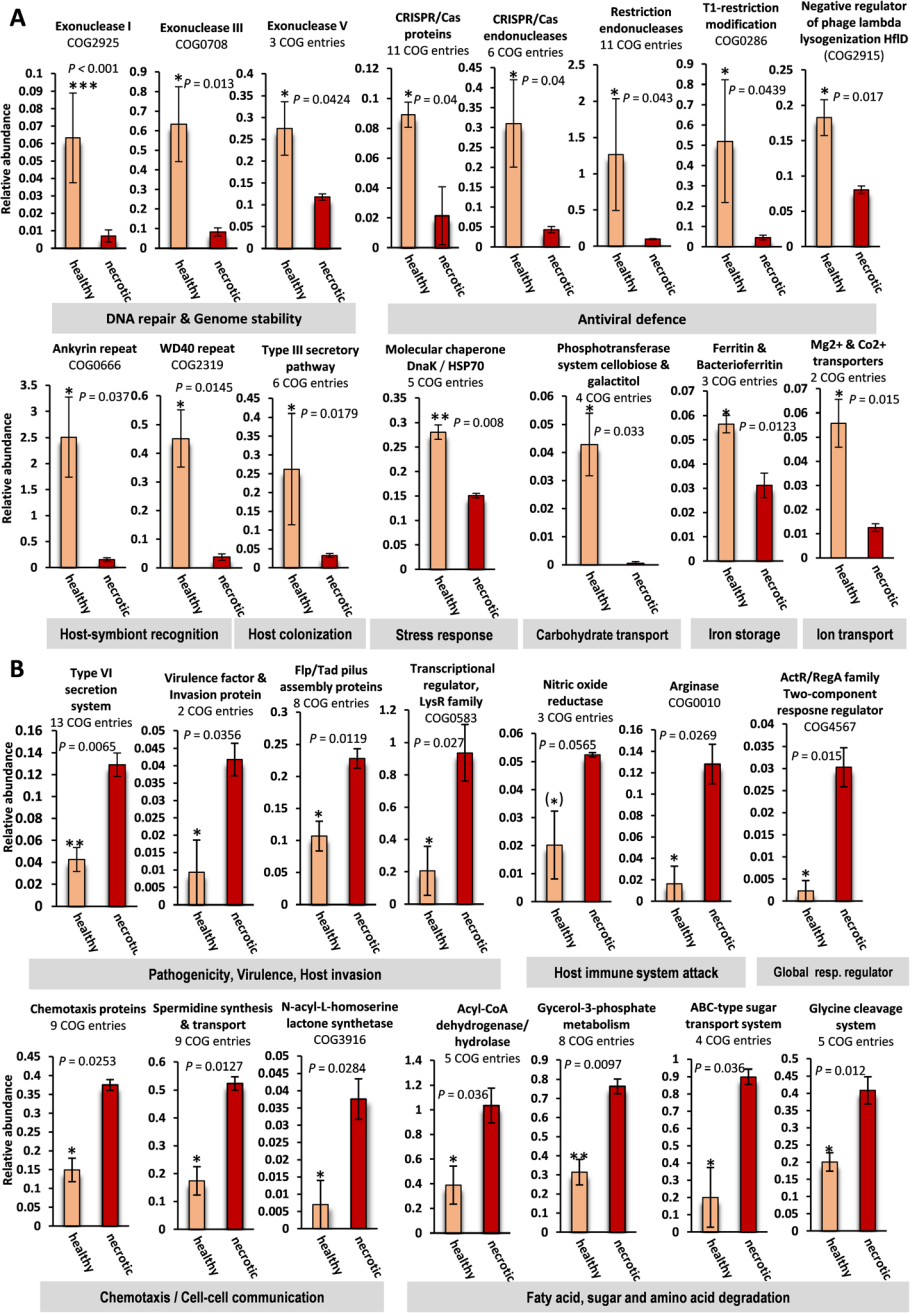
Gene functions which are enriched in the microbiomes of healthy or necrotic *Eunicella gazella* tissue. Average proportions (%) ± standard errors of Clusters of Orthologous Groups of proteins (COGs) that are significantly augmented in the microbiomes of healthy (**a**) or necrotic (**b**) *Eunicella gazella* tissue. If a given function was represented by more than one COG entry across the dataset, the proportions of these functionally belonging COGs were summed, and the number of COGs that contributed to each bar chart is given below chart titles (see Additional file 2: Table S7(C) for the COG entries used). If only one COG entry contributed to a chart, the respective COG ID is given. Paired *t*-tests were used to check for significant differences between sample groups. Statistical significance was established at *P*-values ≤ 0.05. Asterisks above error bars indicate significant differences (*P ≤ 0.05; **P ≤ 0.01; ***P ≤ 0.001). Respective *P-*values are presented in the graphs

To identify the functional signatures of the prokaryotic communities in healthy octocoral tissue, we analyzed the COG entries that showed overall significant differences between sample groups based on ANOVA tests performed with the metagenomics analyses pipeline MGAP of the IMG/M platform from DOE-JGI. Here, we focused on the COGs that were particularly enriched in healthy in comparison with necrotic *E. gazella* samples (Additional file 2: Table S7a-c). Abundances of exonucleases of types I, III, and V involved in DNA repair and genome stability were higher in the prokaryotic communities of healthy *E. gazella* tissue (Fig. 4a) while much less abundant in necrotic tissue. The prokaryotic communities of healthy tissue were also significantly enriched in a large number of COGs related to antiviral defense. These included multiple COGs related to restriction endonucleases, clustered regularly interspaced short palindromic repeats (CRISPR)/CAS proteins (e.g., Cmr3 to Cmr6, Csh2), and CRISPR/CAS endonucleases (e.g., Cas1, Cas2, Cas3), as well as two COGs related to a T1 restriction modification system and a negative regulator of phage lysogenization, respectively. Another feature of the prokaryotic communities of healthy *E. gazella* tissue were functions encoding for eukaryotic-like proteins (ELPs), such as ankyrin and WD40 repeats, which are important for host-symbiont recognition. COGs related to the type III secretory pathway, involved in host colonization, were found at higher abundance in the prokaryotic communities of healthy tissue. The ability to respond to heat and/or heavy metal stress was another characteristic of the healthy octocoral microbiome, evidenced by an increased abundance of genes encoding for molecular chaperones, particularly the bacterial heat-shock-protein DnaK (COG0443; also known as HSP70). Genes involved in carbohydrate cycling and micronutrient acquisition, transport, and storage were another signature of the prokaryotic community of healthy *E. gazella* tissue. We found several COGs related with phosphotransferase systems that facilitate the import of sugar dimers and monomers resulting from polysaccharide breakdown into the cell. Moreover, genes encoding for magnesium and cobalt transporters and the iron storage proteins ferritin and bacterioferritin were significantly enriched in the healthy octocoral tissue.

All these functions were not only greatly reduced in the microbiome of necrotic *E. gazella* tissue but also in the surrounding seawater (Additional file 1: Figure S4a). By contrast, the microbiomes from healthy *E. verrucosa* and *L. sarmentosa* tissue followed a pattern similar to that observed in the healthy *E. gazella* samples. However, while the relative abundances of the abovementioned gene functions in *E. verrucosa* and *E. gazella* were highly similar, in *L. sarmentosa*, some functions (e.g., exonuclease III and CRISPR/Cas endonucleases) tended to be of somewhat lower abundance (Additional file 1: Figure S4a).

### Functional signatures of prokaryotic communities in necrotic octocoral tissue

We found many significantly enriched functions in necrotic *E. gazella* tissue (when compared to healthy tissue samples) that are related to pathogenicity, virulence, and host invasion. These included 13 COG functions linked to the type VI secretion system and eight COGs affiliated with Flp/Tad pilus assembly proteins, two COGs linked to the virulence factor B protein, and the invasion protein IalB and a LysR type transcriptional regulator (Fig. 4b). The prokaryotic communities of necrotic *E. gazella* tissue were further defined by an increased abundance of COGs related to chemotaxis protein synthesis, spermidine (polyamine) synthesis and transport, and *N-*acetyl-L-homoserine lactone production. We also found nitric oxide reductase and arginase-encoding genes enriched in necrotic *E. gazella* samples, which help bacterial pathogens to overcome the host’s immune response. Moreover, the two-component response regulatory system of the ActR/RegA family—characteristic of *Rhodobacteraceae* bacteria—was also enriched in necrotic tissue samples. Finally, efficient fatty acid, sugar, and amino acid breakdown characterized the prokaryotic communities of necrotic octocoral tissue, as evidenced by the increased abundance of over 20 COG entries associated with acyl-CoA dehydrogenases, glycerol-3-phosphate metabolism, ABC-type sugar transport, and glycine cleavage systems. This suggests a feasting of the microbial community on the compromised tissue. Many of these pathogenicity-related functions were significantly enriched in necrotic samples in comparison with all other surveyed microbiomes **(**Fig. 4, Additional file 1: Figure S4 b, c).

### Prokaryotic communities in octocorals and seawater encode for distinct secondary metabolisms

**Fig. 5:**
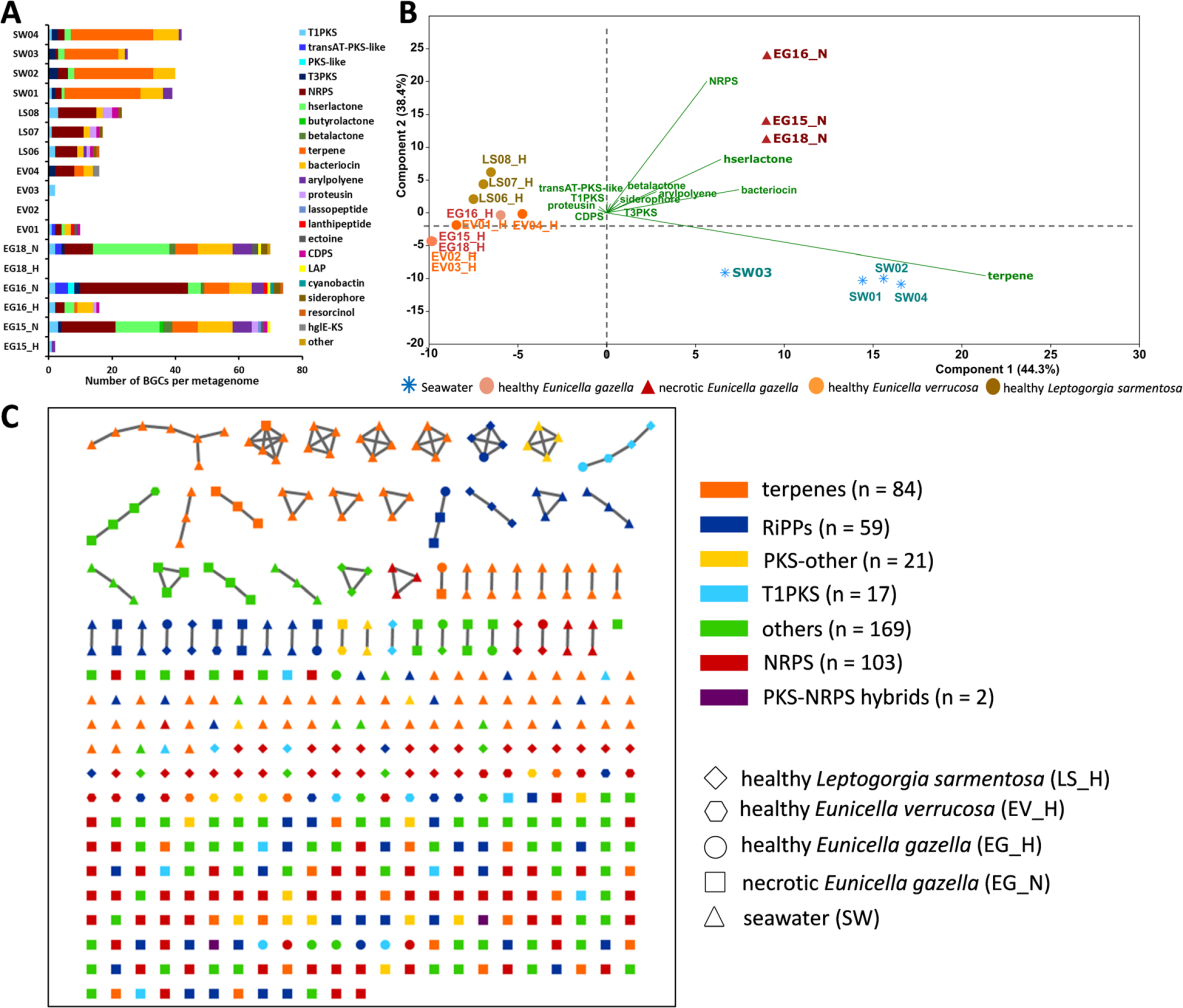
Secondary metabolite coding potential in the prokaryotic communities of healthy and necrotic octocoral samples and seawater. **a** Distribution of biosynthetic gene clusters (BGCs, *N* = 462) across these 17 assembled, metagenomes. The BGC counts per compound class were obtained using antiSMASH version 5.0. **b** Principal component analysis (PCA) biplot based on the BGCs (arrows) found in the metagenomes of the microbiomes of healthy (EG_H, salmon circles) and necrotic (red triangles; EG_N) *Eunicella gazella* tissue, healthy *Eunicella verrucosa* (orange circles; EV01 - EV04), healthy *Leptogorgia sarmentosa* (olive circles; LS06 - LS08), and seawater (blue asterisks; SW01 - SW04). **c** Similarity network of 455 BGCs predicted by antiSMASH and grouped into biosynthetic gene cluster families (GCFs) across seven major compound classes using the BiG-SCAPE algorithm. The network was rendered in Cytoscape. Nodes represent amino acid sequences of BGC domains, and their different shapes indicate the origin of the sampled metagenome. BGC classes are color-coded with number of GCFs per class given in brackets. A majority of the GCFs (“Others,” *N* = 169) could not be classified using current BiG-SCAPE BGC nomenclature. Most GCFs were composed by only one BGC (singletons), with GCFs containing two or more BGCs represented through BGC networks inferred from protein sequence homology. PKS, polyketide synthase; NRPS, non-ribosomal peptide synthetase; RiPPs, ribosomally synthesized and post-translationally modified peptides; hserlactone, homoserine lactone; CDPS, tRNA-dependent cyclodipeptide synthases; hglE-KS, heterocyst glycolipid synthase-like PKS; LAP, linear azol(in)e-containing peptides

AntiSMASH analyses predicted 462 secondary metabolite BGCs within the 17 assembled metagenomes, although most BGCs were located at contig edges and are incomplete. The five most dominant BGCs in the dataset belonged to terpenes (119), followed by NRPS/NRPS-like (108), bacteriocins (69), homoserine lactones (53), and T1PKS (21). The highest number of BGCs was found in necrotic octocoral samples with ≥70 clusters per metagenome, followed by seawater samples with up to 42 clusters per metagenome and healthy *L. sarmentosa* with up to 23 clusters. Healthy *Eunicella* samples had a low number of BGCs, ranging from 0 to 16. Seawater samples were largely dominated by terpene BGCs; several of which were related to carotenoids, followed by bacteriocin, T3PKS, and NRPS clusters. Necrotic *E. gazella* samples possessed the highest diversity of BGCs, which were consistently identified in each replicate sample, as NRPS, bacteriocin, homoserine lactone, aryl-polyene, and terpene BGCs (Fig. 5a, Additional file 2: Table S8a). The metagenomes of healthy *L. sarmentosa* were defined predominantly by NRPS and ribosomally synthesized and post-translationally modified peptide (RiPPs; mainly proteusin) BGCs, while *Eunicella* samples harbored various polyketide synthase BGCs, mainly of “type 1”. Corroborating ordination analyses of taxonomic and functional profiles (Fig. 2), principal components analysis (PCA) confirmed a separate grouping of samples into three distinct clusters (healthy octocorals, necrotic *E. gazella*, and seawater) according to their BGC profiles, with high statistical support (PERMANOVA: *F* = 22.04, *P* = 0.0001; Fig. 5b). Only 13% (60 in 462) of the predicted BGCs showed some level of homology with known BGCs publicly available in the MIBig database, with 31 (7%) BGCs having a similarity value above or equal to 60% (Additional file 2: Table S8b, c).

The BiG-SCAPE algorithm grouped the BGCs predicted with antiSMASH into 103 NRPS, 84 terpene, 59 RiPPs (which include bacteriocins and lantipeptides), 21 “PKS-other,” 17 type I PKS, two PKS-NRPS hybrids, and 169 “other” and non-classifiable “Gene Cluster Families” (GCFs) (Fig. 5c, Additional file 2: Table S9). Most of these families included only a single BGC. GCFs containing two or more BGCs were found mainly among terpenes, followed by RiPPs and other, non-classifiable clusters. Although quite abundant in the octocoral microbial metagenomes, NRPS clusters were mostly unrelated singletons, suggestive of an unprecedented high diversity of NRPS present in these host-associated microbiomes. Congruent with trends observed via ordination analysis, most GCFs with more than one BGC appeared to be habitat specific rather than shared between sample types. For example, the majority of terpene GCFs with two or more BGCs exclusively contained seawater-derived BGCs while the T1-PKS GCFs with more than one BGC were only present in healthy samples of all three octocoral species (Fig. 5c).

## Discussion

This study is the first cultivation-independent, shotgun metagenome-based survey of the taxonomic and functional features of prokaryotic communities in octocorals and its surrounding environments. Our comparative analyses revealed many distinct, adaptive traits of the healthy octocoral holobiont, and intricate shifts in functional and taxonomic structures which underpin a state of dysbiosis of the octocoral microbiome. These findings were accompanied by a thorough description of environmental prokaryotic communities, allowing us to address the connectivity between microbial taxa and functions across healthy and necrotic octocoral tissue, seawater, and sediments. Our multi-layered approach encompassed comparisons of taxonomic (16S rRNA gene), functional (COGs and Pfams), and secondary metabolic (BGCs) profiles, all of which support the notion of highly divergent prokaryotic community structures across all sample categories here examined.

### Unclassified and uncultured *Oceanospirillales* and *Alteromonadales* taxa characterize the temperate octocoral microbiome

Some species of the orders *Alteromonadales* and *Oceanospirillales* are known to engage in mutualistic interactions with their hosts. *Pseudoalteromonas porphyrae*, for example, is a marine alga symbiont known to promote plant growth by stimulating germination and shoot growth through catalase production [42]. Another *Pseudoalteromonas* sp. strain, OT59, isolated from a healthy specimen of the octocoral *Leptogorgia alba* produces the polyketide alteramide A, a compound that displays light-dependent antifungal activity [26]. In the darkness, the strain produces larger quantities of alteramide A which inhibits the growth of the putative coral pathogen *Penicillium citrinum*, implying that coral-associated bacteria could protect their host from infections during heterotrophic night feeding when coral polyps are most exposed. Moreover, coral-associated *Pseudoalteromonas* spp. were recently shown to function as coral probiotics as part of a “beneficial microbes for corals” (BMCs) consortium that was able to reduce coral bleaching under temperature and pathogen challenges in aquarium experiments [43].

High abundances of *Gammaproteobacteria*, comprising specific *Endozoicomonadaceae* and other *Oceanospirillales*, *Alteromonadales*, and *Cellvibrionales* phylotypes, were found in this study to be characteristic of healthy octocoral tissue. Previous surveys recognized the dominance of *Endozoicomonas*-like phylotypes in many coral-bacterial assemblages, particularly in temperate octocorals and *Eunicella* species [8–13, 16]. Yet here we reveal that the structure and dynamics of the gammaproteobacterial consortium associated with healthy corals may be more complex than previously thought. In agreement with van de Water et al. [10], we observed that these typical *Gammaproteobacteria* phylotypes may shift in proportions across different host genera (*Leptogorgia* vs. *Eunicella*) and greatly reduce in abundance during the transition from healthy to necrotic states. Several of the phylotypes that we found significantly enriched in healthy *E. gazella* can be considered core members of the microbiome of temperate gorgonian corals as they were found in all tested healthy samples of the three octocoral species, complying with the “core microbiome” definition suggested by van Oppen and Blackall [44].

Our primer-less, metagenomic-centered approach revealed that among the top 10 most distinctive phylotypes enriched in healthy tissue, only two corresponded to cultured, described species of the genera *Pseudoalteromonas* and *Haliea*, respectively. The other seven *Gammaproteobacteria* on that top-ten list were not classifiable at genus or even family level. These phylotypes, although belonging to a bacterial class well represented by a high diversity of cultivatable species, are likely obligatory symbionts whose growth requirements are difficult to meet under standard laboratory conditions. Moreover, beyond being thus far uncultivatable, these *Gammaproteobacteria* spp. are likely missed by amplicon sequencing strategies which are commonly used in the description of coral microbiome diversity. For example, the unclassified *Oceanospirillaceae* and *Alteromonadales* phylotypes as well as the OM60 and 211ds20 families (*Alteromonadales*), found in this study to be significantly enriched in healthy octocoral tissue samples, were not detected in our earlier amplicon-based study of the closely related octocoral *E. labiata* sampled at the same location [9]. In that previous study, the most abundant OTUs more often belonged to well-known, culturable genera such as *Shewanella*, *Vibrio*, or *Ruegeria* [9]. This emphasizes the importance of primer-less, metagenomics tools in the study of microbiome structuring and functioning in nature as to avoid cultivation and primer bias. Finally, both our taxonomic and functional analyses point towards a correlation of octocoral microbiome structure with host phylogeny. This trend is commonly referred to as “phylosymbiosis” which, in this study, was detectable at the genus—but not species—level owing to the distinctive features of *Leptogorgia* and *Eunicella* prokaryotic communities (Additional file 1: extended discussion).

### Complex structural shifts characterize dysbiosis of the octocoral microbiome

Despite a lower alpha-diversity (suggestive of host-driven control of microbiome assembly in the healthy state), surprisingly, we found a higher beta-diversity (i.e., dispersal in community structure among samples) in healthy octocoral tissue as compared to necrotic *E. gazella* tissue, both at the taxonomic and functional gene content level. This would contrast the lately postulated “Anna Karenina principle,” which argues that dysbiotic individuals vary more in microbial community composition than healthy individuals since the microbiological changes induced by many perturbations are stochastic, leading to unstable community states [45, 46]. Yet lower community similarity between healthy scleractinian corals was also reported in a recent 16S amplicon sequencing study on a new “grey-patch” disease [47]. The higher beta-diversity observed in the study of Sweet et al. [47] and in this study may be accounted for because of the long lifespans of many corals which result in structural adjustments of their microbiome to maintain holobiont fitness under changing micro-environmental conditions. Alternatively, our results suggest that the process of necrosis of the octocoral tissue may follow deterministic succession events, leading to convergent community structures across different individuals. We speculate that this might be the case particularly for late stages of necrosis, after the starting decaying tissue had been firstly colonized by eventual opportunists in early successional stages. However, further studies, involving more octocoral species and more replicate samples per health condition, are necessary to fully understand beta-diversity trends in healthy and diseased coral colonies.

As a clear sign of “dysbiosis” [44] and community destabilization, we found significant changes in microbiome composition correlated with the necrotic tissue of *E. gazella.* Such changes were evidenced by a pronounced decrease in abundance of the multiple *Gammaproteobacteria* taxa mentioned above with a simultaneous, significant increase in *Rhodobacteraceae*, *Flavobacteriaceae*, and *Verrucomicrobiaceae* phylotypes. The latter reached relative abundances similar to the ones found in seawater, pointing to an intrusion of typical seawater taxa into the necrotic octocoral tissue. Increased abundances of opportunistic *Rhodobacteraceae* and/or *Flavobacteriaceae* were previously reported for hard corals at anthropogenically impacted sites, under climate change stressors [48, 49] and associated with white band [50] and grey patch disease [47]. However, in contrast to previous, bacterial cultivation-based investigations of temperate octocorals which suggested an infectious disease caused by *Vibrio* pathogens as the necrosis-causing agent [31, 32], our cultivation-independent, primer-less metagenomics approach did not show increased *Vibrio* abundances in the microbiomes of necrotic *E. gazella* tissue when compared to healthy tissue. Rather, dysbiosis in *E. gazella* involves complex structural changes and abundance shifts of bacterial populations. In agreement with previous findings [20, 51], we therefore advocate for a re-evaluation of the “*Vibrio*-paradigm” in future studies. These may combine molecular and cultivation-based detection of microbial pathogens from diseased octocoral tissue to assess cause-effect relationships using infection experiments in microcosms. It is now known that regular cultivation procedures usually favor the growth of low abundance, but rather opportunistic, bacteria from complex microbiomes as divergent as those from soils [52] and marine sponges [38, 53]. Our data suggest that a similar pattern is likely to occur for fast-growing, cultivatable *Vibrio* spp. from coral microbiomes. Their eventual absence or low representation in amplicon-sequencing datasets [34] may not necessarily derive from primer bias, but from the fact that they are simply low abundant across several animal-microbe associations as suggested elsewhere for fish [54] and marine sponges [53]. Future surveys on different host species, sampling locations, and seasons will shed light on whether the pattern of dysbiosis observed here is universal across coral-microbiome associations.

### DNA stabilization mechanisms and antiviral defense are hallmarks of octocoral-microbiome symbiosis

Our study shows that the functional profiles of prokaryotic communities in healthy tissue of the three octocoral species (albeit quite variable within *Eunicella* specimens) were distinctive from necrotic octocoral tissue and seawater. Exonucleases I, III, and V, found at higher abundance in healthy samples of the three octocoral species, have been thoroughly characterized in the model bacterium *Escherichia coli* [55]. Exonucleases I and III are typically involved in dsDNA repair and homologous recombination, including the repair of oxidative and ionizing radiation damage, which may contribute to a higher plasticity of the microbiome of healthy octocorals to respond to environmental stressors. In addition, the highly processive exonuclease V possesses also an endonuclease function which restricts bacteriophage infections [55]. Indeed, we here reveal for the first time that the prokaryotic consortium of healthy octocorals maintains a sophisticated antiviral defense arsenal, comprising not only the type V exonuclease but also multiple restriction endonucleases together with a complex CRISPR/Cas system and phage lysogenization regulators. Since an enrichment of CRISPR/Cas endonucleases was common to all specimens of the three octocoral species studied here, we argue that CRISPR-associated proteins are a true hallmark of the healthy, temperate octocoral microbiome. The CRISPR/Cas machinery functions as an efficient prokaryotic immune system that transmits resistance to bacteriophages and other foreign genetic elements such as plasmids [56], permitting bacterial survival within a densely populated holobiont. A symbiotic community well protected from viral infections and foreign DNA that is brought in through the suspension-feeding activity of the octocoral polyps is likely more stable, and such improved defense mechanisms may be a prerequisite for lasting interactions with the host. Although undescribed in octocorals, a higher incidence of CRISPR-Cas and restriction endonucleases has been reported in earlier metagenomics surveys on the microbiomes of healthy, marine sponge specimens [57, 58]. Recent analyses of viromes in hard corals revealed lower viral loads in these hosts than in seawater in spite of higher bacterial abundances in the coral host. This suggests a prevailing lysogenic, instead of lytic, mode of interaction between coral symbionts and their bacteriophages [59, 60]. However, a sophisticated CRISPR/Cas defense system and potential prophage was also identified on the genomes of two *Cyanobacteria* species isolated from black band diseased coral [61]. Future studies of the octocoral virome hold promise in revealing the role of bacteriophages in mediating host-microbe interactions within these animals.

### High abundances of eukaryotic-like repeat protein-encoding genes may facilitate host-symbiont recognition in the octocoral holobiont

The prevalence of eukaryotic-like proteins (ELPs), namely ankyrin and WD40 repeats, likely underpins the mechanisms through which host-symbiont recognition and a stable partnership with the host are enabled within octocorals. Ankyrin repeats were by far the most abundant type of ELPs in the healthy *E. gazella* microbiome, and their role in preventing phagocytosis of bacteria by eukaryotic cells has been documented experimentally [62]. Given their presumed eukaryotic origin, the presence of ELPs in bacterial genomes is interpreted as the result of lateral host-microbe gene transfer. The establishment of mutualistic interactions also requires the suppression of local immune responses to allow successful colonization of the host. Although type III secretion systems have primarily received attention due to their role in host colonization by pathogenic bacteria, there is growing evidence that beneficial bacteria also use this system to secrete immune-suppressive effectors into host cells to facilitate host colonization [63]. The enrichment of type III secretion system encoding genes in the microbiomes of healthy octocoral tissue observed in this study supports this notion.

### The octocoral microbiome is enriched in abiotic stress response mechanisms

As a likely adaptative response to heat, oxidative, and/or other abiotic stressors, we found an enrichment of genes encoding for the molecular chaperone DnaK (HSP70) in the prokaryotic consortium of healthy tissue from all three octocoral species. HSP70-like heat shock proteins are ubiquitous in nature, present in most if not all micro- and macro-organisms and have been reported to be highly expressed, for example, by the marine bacterium *Vibrio harveyi* as well as the stony coral *Stylophora pistillata *in response to in vivo heat exposure [64, 65]*.* This finding suggests plasticity of the octocoral microbiome to respond to environmental stressors. We also found evidence that the prokaryotic community in healthy *E. gazella* tissue could contribute with specific micronutrients to the holobiont, such as iron, cobalt, and magnesium. The latter are essential cofactors for numerous enzymes. Cobalt, for example, is an essential factor of vitamin B12 (cobalamin) which is required by eukaryotes for growth and synthesized by gastric cavity bacteria in the semi-closed gastrovascular system of corals [17]. The supply of B vitamins, among other nutrients, was also a key feature identified on the metagenome-assembled genomes of bacterial symbionts from the scleractinian coral *Porites lutea* [66]*.* Indeed, biosynthesis pathways for cobalamin (B12) and other B vitamins are also a common feature of alphaproteobacterial symbionts of marine sponges [38], suggesting that these pathways could be generally important for marine invertebrate growth.

### Pathogenicity features and potential to silence the host immune response revealed for prokaryotic assemblages in necrotic octocoral tissue

Many metabolic features related to macronutrition, particularly lipid, amino acid, and sugar degradation, were enriched in necrotic octocoral tissue. This suggests an enhanced catabolic capacity of the prokaryotic consortium from necrotic *E. gazella* samples to consume the dying octocoral tissue and organic matter made available. The necrotic tissue was largely characterized by pathogenic features that facilitate host invasion, virulence, and host immune system attack, which are possibly tightly regulated by quorum-sensing mechanisms, as evidenced by an increased abundance of homoserine lactone synthetase-encoding genes.

This opportunistic community of prokaryotes further shows the ability to overcome attacks by the host’s immune system by quenching its nitric oxide (NO) production through nitric oxide reductase and arginase activity. NO production is a typical cellular stress response in cnidarians that attempts to kill pathogens with a nitric oxide burst [67]. Oxidative stress has been reported as a driver of scleractinian coral-*Symbiodiniaceae* mutualism breakdown, and components of the integrated stress response pathway, including reactive species such as NO, have been extensively detected in coral holobionts as stress responses to warm water and infection [67, 68]. Yet several pathogens possess sophisticated mechanisms to counteract NO production [69]. A crucial amino acid that modulates the cellular immune response during infection is arginine, which is the common substrate for both inducible nitric oxide synthase (iNOS) and arginase, as well as spermidine production. Generation of NO from arginine is responsible for efficient immune response and cytotoxicity of host cells to kill invading pathogens. But the conversion of arginine to ornithine and urea via the arginase pathway drains the substrate necessary for NO production from the host and supports the growth of bacterial pathogens [69]. Competition between iNOS and arginase for arginine can thus contribute to the outcome of several bacterial infections. Moreover, high arginase activity will result in ornithine production, an essential substrate for the polyamine pathway, leading to putrescine and spermidine synthesis [70]. In line with this, we found multiple genes related to spermidine synthesis and transport enriched in the metagenomes of necrotic tissue. Spermidine has multiple functions in bacterial pathogenicity, including the induction of host cell apoptosis, biofilm formation, escape from phagolysosomes, bacteriocin production, toxin activity, and protection from oxidative and acid stress [71]. Future research shall invest in linking function with taxonomy through the analysis of metagenome-assembled genomes (MAG) to associate the pathogenic features and virulence factors described here with specific, putative coral pathogens.

### Differential natural product biosynthesis capacities revealed for octocoral and seawater microbiomes

Octocorals are prolific sources of natural products with fascinating and unusual chemical structures and bioactivities of interest to medicine and biotechnology. Despite growing evidence from culture-based studies for an existing role of microbial associates in the production of some of these bioactive compounds [28], a mechanistic understanding of the contribution of the octocoral microbiome (including uncultured symbionts) to the chemical diversity of octocorals is lacking. In this study, we illuminate the natural product biosynthesis potential within octocoral-associated and free-living prokaryotic communities and show higher numbers and diversity of natural product BGCs in the metagenomes of necrotic octocoral tissue than in healthy tissue and seawater. Our results suggest that bacterial colonizers of necrotic octocoral tissue are well equipped to compete with resident microbes and to damage the host-tissue through antimicrobial compounds and toxin production. However, we also found a high diversity of NRPS and RiPPs encoding BGCs in the prokaryotic metagenomes of healthy *L. sarmentosa* specimens, while seawater communities were shown to be prolific sources of diverse and novel terpene BGCs. Intriguingly, within each compound class, these BGCs were mostly unrelated with one another, possessing dissimilar gene composition and architecture (i.e., synteny), and did not show significant similarity to known BGCs in public databases. This highlights the amount of genetic novelty likely present in both free-living and host-associated marine microbiomes and pinpoints the *L. sarmentosa* holobiont and bacterioplankton prokaryotes as possible targets for future bioprospection studies.

Several of the BGCs found in necrotic tissue belonged to *Flavobacteriaceae* and *Rhodobacteraceae* which are phylotypes significantly enriched in these samples. For example, an aryl polyene/resorcinol gene cluster from necrotic tissue samples shared a high degree of similarity with flexirubin, a pigment typical of *Flavobacteria* species, displaying highest homology to the flexirubin-encoding BGC of *Flavivirga*. We further identified a BGC encoding for the siderophore bisucaberin B in necrotic tissue samples with a closest sequence match to *Aquimarina*, another *Flavobacteriaceae* genus found in increased abundance in necrotic octocoral tissue. Bisucaberin B has previously been isolated from the sponge-associated, closely related flavobacterium *Tenacibaculum mesophilum* [72].

Some of the BGCs detected in the microbiomes of healthy octocoral tissue were from the enriched *Gammaproteobacteria* fraction (e.g., to the genera *Pseudoalteromonas* and *Shewanella* in the order *Alteromonadales*), indicating that these microbial symbionts could play a role in the chemical defense of their host or compete with other microorganisms within the octocoral holobiont. Some of the NRPS clusters detected in the microbial metagenomes of necrotic and healthy octocoral tissues encode for compounds with known antimicrobial activity. For example, in healthy *L. sarmentosa* samples, we found a BGC encoding for the antifungal compound bicornutin, a compound originally isolated from symbiotic *Xenorhabdus* (*Gammaproteobacteria*) [73], and a BGC encoding for the antibacterial, cyclic lipopeptide rhizomide, originally found in *Burkholderiales* (*Betaproteobacteria*) [74]. The latter, putative rhizomide BGC showed sequence similarity to *Pseudoalteromonas phenolica*, a marine species recognized for its antibacterial activity [75]. Finally, in the microbial metagenomes of necrotic octocoral tissues, we further detected the NRPS clusters of the broad-spectrum antibiotic and lipopeptide paenibacterin [76] and the cytotoxic compound luminmide [77], most similar to the luminmide BGC of the flavobacterium *Kordia* sp. SMS9.

Together, our results support the hypothesis that selective processes are in place which shape a well-defined chemical repertoire in octocoral and seawater microbiomes, congruent with the patterns of taxonomic (16S rRNA gene profiles) and functional divergence (COG and Pfam profiles) observed in these communities. This indicates that the secondary metabolism of marine microbiomes is largely determined by the composition of their constituent members.

## Conclusion

This study unveils unique and distinctive taxonomic, functional, and secondary metabolic traits of the octocoral microbiome. We uncover several dozens of so-far uncultured *Gammaproteobacteria* phylotypes within the orders *Oceanospirillales* and *Alteromonadales*, among others, which collectively form the microbial core of the holobiont and are likely hallmarks of gorgonian coral health.

Dysbiosis of the octocoral microbiome results from complex structural shifts in the microbiome whereby multiple opportunistic and potentially pathogenic taxa are involved in the progression of tissue necrosis, severely reducing the abundance of mutualistic symbionts in the emerging, necrotic state. Contrary to conventional assumptions on the participation of *Vibrio* spp. in coral disease development, members of the *Flavobacteriaceae* (e.g., *Flavobacterium*, *Aquimarina*, and *Tenacibaculum*) and *Rhodobacteraceae* families (e.g., *Loktanella* and *Octadecabacter*), typical seawater opportunists, rank among the most evident microbial indicators of octocoral dysbiosis.

Antiviral defense (enrichment in CRISPR-Cas systems and endonucleases), micronutrient acquisition, and heat-stress response mechanisms, as well as unique host-microbiome molecular interactions mediated by ELPs, are highlighted here for the first time as keystone functional signatures of octocorals. We argue that these are beneficial microbial traits which can guide future coral reef conservation through microbiome therapy. We also shed new light on the processes by which opportunistic pathogens may overcome the host’s immune system (particularly iNOS), an observation that warrants further investigations into the molecular mechanisms of pathogen attack and disease progression in octocorals and eukaryotic hosts in general.

## Methods

### Octocoral, seawater, and sediment sampling

Sampling took place by scuba diving at ca. 17-m depth on June 17, 2014, in the Atlantic Ocean off the coast of Faro, Algarve, Portugal (“Pedra da Greta”: Lat. 36° 58′ 47.2N, Long. 7° 59′ 20.8W). Bottom water pH was 8.13, temperature 19 °C, and salinity 36.41 ppt.

Branches (10–20 cm each) of 10 colonies from three octocoral (Alcyonacea, Gorgoniidae) species, *Eunicella gazella* (*N* = 6: 3× healthy, 3× necrotic; EG15H, EG15N; EG16H, EG16N; EG18H, EG18N; Additional file 1: Figure S1a,b), *Eunicella verrucosa* (*N* = 4; EV01 - EV04; Additional file 1: Figure S1c), and *Leptogorgia sarmentosa* (*N* = 3; LS06 - LS08; Additional file 1: Figure S1d), were sampled. From *E. gazella*, branches of both healthy (H) and necrotic (N) tissue were sampled from the same colonies which displayed both conditions to compare the octocoral-associated microbial community in healthy versus “diseased” states. Care was taken to collect the healthy and necrotic tissue samples from different branches of the same colony, whereby most of the colony was still in a healthy state, and healthy samples were only taken from a macroscopically entirely healthy-looking branch. The “diseased” tissue from *E. gazella* (hereafter termed “necrotic octocoral tissue” unless stated otherwise) manifested itself in a change of tissue color (from white to brown) and integrity which indicates necrosis and ultimately leads to coenenchyme detachment and loss.

The three octocoral species are commonly found in the Algarve region [6], and specimens of each species were identified in the field based on color and macroscopic, morphological criteria: *E. verrucosa* — colony color from white to cream/beige, heterogeneous surface with “bumps” (verrucae); *E. gazella*—colonies always white with orange polyps, diameter of the ramets noticeably larger than in *E. verrucosa*, colony surface homogeneous with extensive branching mostly in one dimension; *L. sarmentosa*—bush-like colonies growing in one or more dimensions, brick red uniform color, polyps present in all surfaces of the branches [6].

All octocoral samples were placed, *in situ*, separately in Ziploc® plastic bags containing natural seawater. In addition, replicate samples of surrounding seawater (*N* = 4; SW01 - SW04; ca 2 L each) and surface sediment (*N* = 3; SD01 - SD03; ca 100 g each) were collected in separate Ziploc® plastic bags. The samples were transported to the laboratory in a cooling box within 1.5 h post sampling and immediately processed upon arrival as described in [9] and below.

### Sample processing, extraction, and sequencing of total community DNA

Preparation of octocoral and seawater samples for total-community DNA (TC-DNA) extraction followed the procedures described in detail by Keller-Costa et al [9]. Briefly, each octocoral sample was subjected to a differential centrifugation step for the retrieval of microbial cell pellets, which were stored at – 80 °C until TC-DNA extraction. Each seawater sample (2 L; SW01 - SW04) was filtered through a sterile 0.22-μm nitrocellulose membrane filter (Millipore, Billerica, MA, USA; 47 mm) using a vacuum pump, and filters were stored at −80°C until TC-DNA extraction. Per sediment sample (SD01-SD03), 0.5 g was weighed and stored at −80°C until TC-DNA extraction (for further sample processing details, see Additional file 1).

Total community DNA (TC-DNA) was extracted from all 20 samples (4× seawater, 3× sediment, 3× healthy *E. gazella*, 3× necrotic *E. gazella*, 4× healthy *E. verrucosa*, 3× healthy *L. sarmentosa*) with the UltraClean® Soil DNA isolation kit (MO BIO, Carlsbad, CA, USA) according to the manufacturer’s instructions (see Additional file 1 for TC-DNA yields and integrity). Equivalent amounts of TC-DNA per sample category (i.e., octocoral, seawater, sediment) were sent for next generation shotgun sequencing on an Illumina HiSeq 2500 device at MR DNA (Shallowater, Texas, USA). DNA libraries were prepared for sequencing using the Nextera DNA Sample preparation kit (Illumina) after the manufacturer’s instructions and sequenced paired end with sequence depth calibrated at *c.* 20 million 101-bp reads per sample.

### Metagenome data processing and assembly

Unassembled reads were analyzed with the MGnify metagenomics v2.0 platform from the European Bioinformatics Institute (EMBL-EBI) [78] as described earlier [57]. With the MGnify data processing pipeline 2.0, taxonomic and functional profiles of the metagenomes based on 16S rRNA genes (archaeal, bacterial, chloroplast, and mitochondrial operational taxonomic units—OTUs at 97% sequence similarity cutoff levels) and InterPro (IPR) protein domain entries were respectively obtained [78]. In this study, downstream statistical analyses of the unassembled reads focused primarily on the OTU contingency tables delivered using the MGnify pipeline. This enabled us to make optimal use of the full sequencing depth employed in the characterization of all samples, obtaining large amounts of 16S rRNA gene reads to contrast prokaryotic community structure across sediments, seawater, and octocorals. However, we found that the unassembled metagenomes from healthy octocoral tissues contained a significant number of IPR functions typical for multicellular eukaryotes. Hence, we performed the functional analyses of prokaryotic communities on assembled metagenomes after a taxon-specific filtering step (using EukRep, see details below) to remove eukaryotic contigs from the analytical dataset. Assembly was performed using metawrap v1.0.5 [79] and encompassed reads quality control with the metawrap galore module followed by assembly with the metaSPAdes module 3.13.0 [80]. The resulting assemblies were subjected to assignment of eukaryotic contigs with EukRep v.0.6.6 [81]. Eukaryotic contigs were filtered out of the original assemblies, generating individual “prokaryotic-enriched” assembly files per sample. The thus obtained prokaryotic assemblies were uploaded onto the Integrated Microbial Genomes & Microbiomes (IMG/M) system from DOE-JGI [82] and run through the DOE-JGI microbial genome annotation pipeline (MGAP) which performs sequence data pre-processing, feature prediction (CRISPR finder, Infernal, Prodigal, etc.), and functional annotation (COG, Pfam, KEGG, IPR, etc.) and statistics [83]. For functional annotation of metagenomes, we focused on the COG and Pfam outputs, followed by statistical tests (Fisher’s exact test and/or ANOVA with multiple comparison correction and *P*-value adjustment using the Benjamini-Hochberg method) for significant differences among groups performed for the relative abundance of all COG and Pfam entries detected across the data (these tests were executed by MGAP pipeline of the IMG/M platform from DOE-JGI).

### Data analyses and statistics

Data analyses of the OTU contingency table retrieved from the MGnify metagenomics v2.0 platform comprised (i) calculation of symbiont richness and diversity (Shannon’s index) in healthy versus diseased *Eunicella gazella* samples, (ii) assessment of phylum-, class-, and family-level prokaryotic composition in all individual samples, (iii) multivariate analysis of OTU data, and (iv) determination of phylotypes that contributed most to the dissimilarity of the microbiomes of healthy versus diseased octocoral samples. OTUs assigned to chloroplasts (*N* = 15) and mitochondria (*N* = 7) were removed from the dataset. For (i) alpha-diversity comparisons of healthy with necrotic *E. gazella* tissue, the OTU data were rarefied to the least sequenced sample (EG16_H-1702 reads), using the “rarefy_even_depth” function of the “phyloseq” package in R. All other analyses were performed on the full OTU datasets to make optimal use of the full taxonomic information retrieved from the data. For (ii) prokaryote community composition, relative abundance data (percentages) were used. For (iii) multivariate analyses and (iv) comparison of the most differentiating phylotypes, the OTU tables were Hellinger-transformed to normalize the dataset. The Bray-Curtis dissimilarity index was employed as metric to perform principal coordinate analyses (PCoA) of samples according to their OTU profiles (iii), and data were plotted in Eigenvalue scale using PAST v3.25 [84]. PERMANOVAs—permutational multivariate analyses of variance—were performed with 999 permutations to test for overall differences in the Bray-Curtis dissimilarity matrix between sample categories. Multivariate homogeneity of group dispersions (variances) was tested using the betadisper function of the vegan v.2.4-2 package in R. To determine phylotypes that contributed most to the dissimilarity of the microbiomes of healthy versus diseased octocoral samples (iv), similarity percentage analysis (SIMPER) in PAST v 3.25 [84] was performed on Hellinger-transformed abundances. SIMPER assesses the most differentiating taxa among groups of samples based on a ranked dissimilarity matrix, calculated, in this case, using the Bray-Curtis index. In addition, an “Indicspecies” analysis [36, 37] was ran, using the “Indicspecies” v.1.7.9 package in R (function “r.g.”, with *α*=0.1), to assess the strength of the relationship between phylotype occurrence and abundance and the health status of *E. gazella* tissue. Finally, to further test which prokaryotic phylotypes of healthy versus necrotic octocoral tissue varied significantly, paired *t*-tests were performed. One-way ANOVAs followed by Bonferroni *t*-tests were used to check for significant differences of selected OTUs between the healthy octocoral species, sediment, and seawater in Fig. 3. For all those OTUs, normal distribution of residuals was confirmed by Shapiro-Wilk tests and equal variance by Brown-Forsythe tests.

Functional comparisons were carried out using COG and Pfam-based annotations of assembled metagenomes retrieved from the IMG system and comprised (v) multivariate analyses of samples according to their COG and Pfam profiles and (vi) determination of COGs that differed significantly in abundance between the microbiomes of healthy versus diseased octocoral samples. COG and Pfam tables were Hellinger-transformed, and multivariate analyses (v) were performed as described above. To test whether the relative abundances (percentages) of the most differentiating COGs of healthy versus necrotic octocoral tissue varied significantly (vi), paired *t*-tests were executed. Whenever data presented both, normal distribution (assessed by Shapiro-Wilk tests) and equal variance (assessed by Brown-Forsythe tests), one-way ANOVAs followed by Bonferroni *t*-tests were used to test for significant differences of selected COGs between the healthy octocoral species and seawater. If one of the abovementioned assumptions was violated, Kruskal-Wallis tests on ranks were used, followed by Dunn’s post hoc tests.

To gain insights into the secondary metabolite production capacities of the microbiomes of healthy and necrotic octocoral tissues versus seawater (vii), all assembled metagenomes were screened for the presence of secondary metabolite biosynthetic gene clusters (BGCs) using antiSMASH v.5 [85] with a “relaxed” detection strictness and extra features “all on”. AntiSMASH identifies known and putatively novel BGCs underlying the possible biosynthesis of major compound classes such as polyketides, terpenoids, non-ribosomal peptides (NRPs), and beta-lactones, using the MiBiG database [86, 87]. A variance-covariance matrix was calculated based on the abundances of BGCs in the metagenomes. A principal component analysis (PCA) was then performed and an ordination biplot created using PAST v3.25 [84]. Amino acid sequences were then retrieved in GenBank (fasta) format from the antiSMASH results and used as input data for downstream analyses with the “Biosynthetic Genes Similarity Clustering and Prospecting Engine” (BiG-SCAPE) [88] at default settings. This tool allows the construction of similarity networks between the BGCs predicted by antiSMASH, through the use of the Pfam database and the hmmscan algorithm, from the HMMER suite [89], to predict Pfam entries in each sequence [90]. For every pair of BGCs in the set, the overall distance between them is calculated as the weighted combination of their dissimilarity in protein domain content, synteny, copy number, and sequence identity [88]. An upper distance cutoff value of 0.3 was used to define “Gene Cluster Families” (GCFs) for BGCs identified by antiSMASH based on their distances as explained above. The generated network files—separated by BiG-SCAPE classes—were then combined for visualization using Cytoscape version 3.7.2 [91].

## Supplementary Information

**Additional file 1. **Detailed Methodology. Sample processing and total-community DNA (TC-DNA) extraction. Extended Results. Features of the 16S rRNA gene-based taxonomic dataset and of metagenome assemblies subjected to functional analyses. Abundance distributions of bacterial classes across sample categories. Alpha-diversity in octocoral-associated and environmental prokaryotic communities. Extended discussion. Evidence for phylosymbiosis. **Supplementary Figure S1.** Photographs of the octocorals investigated in this study. (a) healthy *Eunicella gazella,* (b) *Eunicella gazella* branches with necrosis signs (black arrows), (c) healthy *Eunicella verrucos*a, (d) healthy *Leptogorgia sarmentosa.* (a, c and d) are underwater photographs of the octocorals in their natural habitat taken off the Algarve coast (South Portugal). **Supplementary Figure S2.** Class-level prokaryotic community profiles of healthy (EG_H) and diseased (EG_N) *Eunicella gazella* tissue, healthy *Eunicella verrucosa* (EV01 - EV04) and *Leptogorgia sarmentosa* (LS06 - LS08) specimens as well as seawater (SW01 - SW04) and sediment samples (SD01 - SD03). Relative abundances are displayed for taxa representing more than 1% of the total dataset reads. Taxa with abundances below 1% across the data are collectively labeled as ‘rare classes’. **Supplementary Figure S3.** Venn diagrams showing the shared and specific prokaryote phylotypes in octocorals and surrounding environments. (a, b) OTUs common and exclusive to seawater (blue), sediment (black) and healthy tissue of the octocorals *Leptogorgia sarmentosa* (olive), *Eunicella verrucosa* (orange) and *E. gazella* (salmon). (c, d) OTUs common and exclusive to seawater (blue), sediment (black) and healthy (salmon) *versus* necrotic (red) tissue of *E. gazella*. (a, c) Replicate samples were pooled to portray the total number of prokaryote OTUs within each sample category. (b, d) Only those OTUs present in all replicate samples of each sample category are shown. The following three OTUs were shared only between all octocoral samples while not consistently detectable in sediment and seawater: OTU_866 *Endozoicomonadaceae*; OTU_805 *Shewanella bentica*; OTU_943 *Pseudoalteromonas porphyrae*. Venn diagrams were created using the online tool InteractiVenn (http://www.interactivenn.net/ [92]). **Supplementary Figure S4.** Abundance distributions of functional features enriched in the microbiomes of either healthy or necrotic  *Eunicella gazella* tissue across the metagenomes of all octocoral species and seawater. Relative abundances of those Cluster of Orthologous Genes (COGs) of proteins in the microbiomes of the different octocoral species and seawater that were shown (in Figure 4) to be significantly enriched in the microbiomes of healthy (a) or necrotic  (b and c) *Eunicella gazella* tissue. Bars represent average proportions (%) ± standard errors. If a given function was represented by more than one COG entry across the dataset, the proportions of these functionally belonging COGs were summed and the number of COGs that contributed to each bar chart is given below chart titles; if only one COG entry contributed to a chart, the respective COG ID is given. If data presented normal distribution (Shapiro-Wilk test) and equal variance (Brown-Forsythe test), One-way-ANOVAs followed by Bonferroni *t*-tests were used to check for significant differences between sample groups. If one of the assumptions was violated, Kruskal-Wallis tests on ranks were instead performed, followed by Dunn’s post-hoc tests. Statistical significance was established at *P*-values ≤ 0.05. Letters above error bars indicate significant differences (*P ≤ 0.05; **P ≤ 0.01; ***P ≤ 0.001). Respective *P-*values are presented in the graphs. Panel (c) is equal to panel (b) but includes the necrotic *E. gazella* samples for an easier visualization of the “fate” of the respective functions in necrotic samples as compared to all healthy octocoral samples and seawater.

**Additional file 2: Supplementary Table S1.** Number of sequence reads per quality control step across all metagenome samples using the MGnify metagenomics pipeline (EMBL-EBI), version 2.0 (project PRJEB13222). **Supplementary Table S2.** Basic information on the assembled metagenome dataset. **Supplementary Table S3.** Number of OTUs and sequence reads per prokaryote phylum across sample categories. **Supplementary Table S4.** (A) Taxonomic assignment and abundance of all prokaryotic OTUs of the dataset, according to the MGnify metagenomics pipeline (EMBL-EBI), version 2.0. (B) Similarity percentage (SIMPER) analyses indicating the contributions of specific operational taxonomic units (OTUs) to observed variation in the prokaryotic community structure between healthy and necrotic octocoral tissue. (C) “Indicspecies” analysis identifying “indicator” OTUs for the healthy and necrotic state of *E. gazella* tissue. **Supplementary Table S5.** General features of the assembled, “prokaryote-enriched” octocoral and seawater metagenomes according to the IMG/M annotation pipeline. **Supplementary Table S6.** Functional profiling of the assembled octocoral and seawater metagenomes (*N* = 17) according to Protein families (Pfam) annotations using the Integrated Microbial Genomes & Microbiomes (IMG/M) system from DOE-JGI. **Supplementary Table S7.** (A) Functional profiling and comparison of the assembled octocoral and seawater metagenomes (*N*=17) according to Clusters of Orthologous Groups of proteins (COGs) annotations using the Integrated Microbial Genomes & Microbiomes (IMG/M) system from DOE-JGI. (B) COG functions respectively enriched in the microbiomes of healthy octocoral tissue (green highlights) and recrotic octocoral tissue (orange highlights) and displayed in Figure 4. (C) Overview of functional features enriched either in the microbiomes of healthy (highlighted in green) or necrotic (highlighted in orange) octocoral tissue according to Clusters of Orthologous Groups of proteins (COGs) annotations using the Integrated Microbial Genomes & Microbiomes (IMG/M) system from DOE-JGI. **Supplementary Table S8.** (A) Secondary metabolite biosynthetic gene clusters (BGCs) predicted for each assembled metagenome (*N* = 17) using antiSMASH v. 5.0. (B) List of biosynthetic gene clusters with some level of homology to MIBiG database entries. (C) Closest taxonomic affiliation (Blastn searches) of biosynthetic gene clusters that shared some level of homology to MIBiG database entries. **Supplementary Table S9.** List of secondary metabolite-encoding biosynthetic gene cluster (BGC) families (GCFs) identified across the data by similarity network analysis using the BiG-SCAPE algorithm.

## Data Availability

All metagenomes were deposited in the European Nucleotide Archive (ENA) under the study accession number PRJEB13222 (ERP014771) and the sample accession numbers SAMEA3913358 (ERS1100492) to SAMEA3913367 (ERS1100501).
