# Supplementary material for: Metagenomic insights into the taxonomy, function, and dysbiosis of prokaryotic communities in octocorals
Source: Microbiome. 2021 Mar 25;9:72. doi: 10.1186/s40168-021-01031-y (PMC7993494; doi:10.1186/s40168-021-01031-y)
Supplement: Supplementary file 2 — Additional file 1. Detailed Methodology. Sample processing and total-community DNA (TC-DNA) extraction. Extended Results. Features of the 16S rRNA gene-based taxonomic dataset and of metagenome assemblies subjected to functional analyses. Abundance distributions of bacterial classes across sample categories. Alpha-diversity in octocoral-associated and environmental prokaryotic communities. Extended discussion. Evidence for phylosymbiosis. Supplementary Figure S1. Photographs of the octocorals investigated in this study. (a) healthy Eunicella gazella, (b) Eunicella gazella branches with necrosis signs (black arrows), (c) healthy Eunicella verrucosa, (d) healthy Leptogorgia sarmentosa. (a, c and d) are underwater photographs of the octocorals in their natural habitat taken off the Algarve coast (South Portugal). Supplementary Figure S2. Class-level prokaryotic community profiles of healthy (EG_H) and diseased (EG_N) Eunicella gazella tissue, healthy Eunicella verrucosa (EV01 - EV04) and Leptogorgia sarmentosa (LS06 - LS08) specimens as well as seawater (SW01 - SW04) and sediment samples (SD01 - SD03). Relative abundances are displayed for taxa representing more than 1% of the total dataset reads. Taxa with abundances below 1% across the data are collectively labeled as ‘rare classes’. Supplementary Figure S3. Venn diagrams showing the shared and specific prokaryote phylotypes in octocorals and surrounding environments. (a, b) OTUs common and exclusive to seawater (blue), sediment (black) and healthy tissue of the octocorals Leptogorgia sarmentosa (olive), Eunicella verrucosa (orange) and E. gazella (salmon). (c, d) OTUs common and exclusive to seawater (blue), sediment (black) and healthy (salmon) versus necrotic (red) tissue of E. gazella. (a, c) Replicate samples were pooled to portray the total number of prokaryote OTUs within each sample category. (b, d) Only those OTUs present in all replicate samples of each sample category are shown. The following three OTUs we [file 40168_2021_1031_MOESM2_ESM.docx]

**Title:**

**Metagenomic insights into the taxonomy, function and dysbiosis of prokaryotic communities in octocorals**

**Running title:** Functional metagenomics of octocorals

**Authors:**

Keller-Costa, T.^1*^, Lago-Lestón, A.^2*^_,_ Saraiva, J.P.^3^, Toscan, R.^3^, Silva, S.G. ^1^, Gonçalves, J.^4^, Cox, C.J.^4^, Kyrpides, N.^5^, Nunes da Rocha, U.^3^ and Costa, R.^1,4,5^

**Affiliations:**

^1^Instituto de Bioengenharia e Biociências (iBB), Instituto Superior Técnico (IST), Universidade de Lisboa, 1049-001 Lisbon, Portugal

^2^Centro de Investigación Científica y de Educación Superior de Ensenada (CICESE), 22860, Ensenada, Mexico

^3^Helmholtz Centre for Environmental Research (UFZ), Leipzig, Germany

^4^Centro de Ciências do Mar (CCMAR), Universidade do Algarve, 8005-139 Faro, Portugal

^5^ Department of Energy, Joint Genome Institute, Walnut Creek, California 94598, USA, and Lawrence Berkeley National Laboratory, Berkeley, California 94720, USA.

**E-mail contacts:**

Keller-Costa, Tina: tinakellercosta@tecnico.ulisboa.pt

Lago-Lestón, Asunción: alago@cicese.mx

Saraiva, João P: joao.saraiva@ufz.de

Toscan, Rodolfo: rodolfo.toscan@ufz.de

Silva, Sandra G.: sandragodinhosilva@tecnico.ulisboa.pt

Gonçalves, Jorge: [jgoncal@ualg.pt](mailto:jgoncal@ualg.pt)

Cox, Cymon J.: cymon@ualg.pt

Kyrpides, Nikos: nckyrpides@lbl.gov

Nunes da Rocha, Ulisses: ulisses.rocha@ufz.de

Costa, Rodrigo: rodrigoscosta@tecnico.ulisboa.pt

***Corresponding authors:**

Instituto de Bioengenharia e Biociências (iBB), Instituto Superior Técnico (IST), Universidade de Lisboa, Av. Rovisco Pais 1, Torre Sul, Piso 11, 11.6.11b, 1049-001 Lisbon, Portugal, Tel: (+351) 21 841 3167, E-mail: [tinakellercosta@tecnico.ulisboa.pt](mailto:tinakellercosta@tecnico.ulisboa.pt). División de Biología Experimental y Aplicada (DBEA), Centro de Investigación Científica y Educación Superior de Ensenada (CICESE), Carr. Ensenada-Tijuana 3918, Zona Playitas, Ensenada C.P 22860, Baja California, Mexico, Tel: (+52) 175 05500, E-mail: [alago@cicese.mx](mailto:alago@cicese.mx).

**Supplementary Material**

*Inventory of the Supplementary Material:*

in this file (Additional file 1):

**Detailed Methodology.** Sample processing and total-community DNA (TC-DNA) extraction.

**Extended Results.** Features of the 16S rRNA gene-based taxonomic dataset and of metagenome assemblies subjected to functional analyses. Abundance distributions of bacterial classes across sample categories. Alpha-diversity in octocoral-associated and environmental prokaryotic communities.

**Extended discussion.** Evidence for phylosymbiosis.

**Supplementary Figure S1.** Photographs of the octocorals investigated in this study.

**Supplementary Figure S2.** Class-level prokaryotic community profiles of healthy and diseased *E. gazella* tissue, healthy *E. verrucosa* and *L. sarmentosa* specimens, seawater and sediment samples.

**Supplementary Figure S3.** Venn diagrams showing the shared and specific prokaryote phylotypes in octocorals and surrounding environments.

**Supplementary Figure S4.** Gene functions which are enriched in the microbiomes of healthy or diseased *Eunicella gazella* tissue.

**Detailed Methodology**

**Sample processing and total-community DNA (TC-DNA) extraction**

Prior to total-community DNA (TC-DNA) extraction, up to 3.2 g of each octocoral sample was rinsed with artificial seawater (ASW), aseptically cut into smaller pieces and the soft tissue (coenenchyme and polyps) was separated from the inner gorgonin skeleton with a scalpel. The soft tissue was homogenized in sterile Ca^2+-^ and Mg^2+^ -free artificial seawater (CMFASW; 1 g of tissue per 9 ml CMFASW) using a sterile mortar and a pestle. The resulting homogenates were centrifuged for 2 min at 500 *g*. The supernatant was transferred into a new centrifuge tube and subjected to a final centrifugation step for 30 min at 10,000 *g*. The resulting octocoral-derived microbial cell pellets were stored at -80°C until total community DNA (TC-DNA) extraction. Each seawater sample (2 L; SW01-SW04) was filtered through a sterile 0.22-μm nitrocellulose membrane filter (Millipore, Billerica, MA, USA; 47 mm) using a vacuum pump. The filters were then aseptically cut into small pieces and stored at -80°C until TC-DNA extraction. Per sediment sample (SD01-SD03), 0.5 g were weighed and stored at -80°C until TC-DNA extraction.

TC-DNA integrity was confirmed by 1.2% agarose gel electrophoresis and concentrations estimated with the Qubit 2.0® dsDNA high-sensitivity assay kit (Life Technologies, Eugene, Oregon, USA). For seawater and sediment samples TC-DNA yield was in the range of ca 800-1300 ng (8 – 13 ng/µL) and for octocoral samples TC-DNA yield was between ca 100-600 ng (0.1 – 0.6 ng /µL).

**Extended Results**

**Features of the 16S rRNA gene-based taxonomic dataset**

The taxonomic analytical dataset consisted of 236,561 prokaryotic 16S rRNA gene reads identified from the data using the MGnify RNA Selector tool. These data were used for taxonomic characterization of all 20 metagenome samples since the 16S rRNA gene read count in the unassembled dataset was significantly higher than the number of 16S reads retrievable from the assembled metagenome data. Nevertheless, the 16S rRNA gene taxonomy retrieved with MGnify (for unassembled data) corroborated the genome-wide based taxonomy retrieved with the Integrated Microbial Genomes & Microbiomes (IMG/M) platform from the Joint Genome Institute (DOE-JGI) for assembled data (see below).

**Features of metagenome assemblies subjected to functional analyses**

Preliminary analyses of the IPR abundance tables obtained from the MGnify metagenomics pipeline on unassembled metagenomes showed that the metagenomes retrieved from healthy octocoral tissue samples were abundant in sequence reads assigned to functions typical for the eukaryotic host and mitochondrion (e.g. cytochrome c and b oxidase, ATPase, F0 complex, NADH dehydrogenase subunits), which were seemingly derived from octocoral DNA. Therefore, to ensure an accurate functional comparison of the microbiomes analysed in this study, all metagenome samples were first assembled into contigs with metaSpades. Contigs of eukaryotic origin were thereafter identified with EukRep [1] and removed from the analytical dataset prior to functional characterisations. Since, however, the metagenome assemblies from the extremely diverse microbial communities of sediment samples were quite poor - yielding only between 185-711 contigs above 1000bp per sample with an average contig length of 1,399 bp -, sediment samples were omitted from further functional analyses.

The total number of contigs with ≥1000bp in length in the 17 octocoral and seawater metagenome assemblies was 381,093 (Additional file 2: **Table S2**). The length of the longest contig in each assembly ranged from 52,627 to 516,565bp. The EukRep [1] pipeline identified 34,939 eukaryotic contigs within the dataset, whereby an average of 10.18% of the contigs were of presumed eukaryotic origin in the microbiomes of healthy octocoral tissue, while the microbiomes of necrotic tissue and seawater had an average of only 0.67% and 2.57% of eukaryotic contigs, respectively. After removal of all eukaryotic contigs, the final prokaryote-enriched assembled metagenome dataset contained a total of 346,154 contigs with ≥1000bp in length (Additional file 2: **Table S2**).

**Abundance distributions of bacterial classes across sample categories**

Despite the decrease in relative abundance of *Proteobacteria* in necrotic octocoral tissue, the number of *Proteobacteria* phylotypes (OTU richness) increased from 177 OTUs (detected across 20,420 total reads) in healthy to 312 OTUs (across 19,927 total reads) in necrotic tissue (non-rarefied libraries, Additional file 2: Table S3), an outcome corroborated by alpha diversity analyses run on rarefied data. Likewise, the class *Gammaproteobacteria* within the *Proteobacteria* phylum dominated the healthy tissues of all host species, but suffered a drastic reduction in relative abundance, by over 50%, within the necrotic tissue samples from *E. gazella* (Additional file 1: Figure S2). Nevertheless, a clear increase in the number of *Gammaproteobacteria* OTUs in necrotic tissue samples was as well observed, following the overall trend described for *Proteobacteria* (Additional file 2: Table S4a). Conversely, a significant increase (*P* = 0.049) in relative abundance was observed for the *Bacteroidetes* phylum, from only 0.6% in healthy to 29% in necrotic octocoral tissue. This was accompanied by an increase in the total number of bacterial phylotypes, from 24 OTUs / 20,420 reads in healthy to 95 OTUs / 19,927 reads in necrotic tissue, placing the abundance and richness of *Bacteroidetes* OTUs in necrotic tissue very close to that of seawater (28% and 85 OTUs detected across 26,039 reads, Additional file 2: Table S3). Notably, the seawater-prevailing classes *Flavobacteriia* (*Bacteroidetes*) and *Alphaproteobacteria* (*Proteobacteria*) both presented higher relative abundances in necrotic than in healthy octocoral tissue samples (Additional file 1: Figure S2).

**Distinct alpha-diversity measures revealed for octocoral-associated and environmental prokaryotic communities**

In total, 1041 prokaryotic OTUs (12 archaeal and 1029 bacterial OTUs defined at 97% 16S rRNA gene similarity) and 93,589 high-quality, bacterial and archaeal reads were obtained from the 20 unassembled metagenome samples (Additional file 2: Table S3). Across the full, non-rarefied dataset, sediment samples displayed the highest number of total OTUs (n = 640 in 10,449 reads), even though less reads were retrieved from sediments in comparison with the subsequent, richest sample categories in the dataset, namely necrotic *Eunicella gazella* samples (n = 550 OTUs in 19,927 reads) and seawater (n = 447 OTUs in 26,039 reads). Overall, lower OTU counts were obtained from healthy octocoral tissues (from 206 OTUs in *L. sarmentosa* to 310 in *E. verrucosa* - Additional file 2: Table S3). All trends above were maintained when alpha diversity analyses were performed on rarefied sequence libraries which, furthermore, clearly confirmed that OTU richness and (Shannon’s) diversity measures were significantly higher (t-test, *P* = 0.001 and *P* = 0.0173) in necrotic than in healthy *E. gazella* tissue.

**Extended discussion**

**Evidence for phylosymbiosis**

We found core prokaryotes present and abundant in all healthy gorgonian samples. However, we observed that, both at the taxonomic and functional levels, there was more overlap between prokaryotic community structures of octocoral specimens of the same genus (*Eunicella*) while more distinct and less dispersed community profiles were detected for *Leptogorgia* specimens. The unique taxonomic features of the *L. sarmentosa* microbiome (e.g. a higher abundance of *Mycoplasmataceae*, *Vibrionaceae*, and unclassified *Oceanospirillales* and *Alteromonadales* phylotypes) in comparison with those of *Eunicella* species, point towards a correlation of octocoral microbial community composition with host phylogeny. Our results are corroborated by similar observations made in a previous 16S rRNA gene based, pyrosequencing study on gorgonian-associated microbial communities in the Mediterranean Sea which included three *Eunicella* species as well as *L. sarmentosa* [2, 3]. Shifts in microbiome composition along with host taxonomy indicate evolutionary divergence in the symbiotic communities. This phenomenon, also known as **‘**phylosymbiosis’, has been repeatedly reported for hexa- and octocoral microbial communities on the taxonomic level [reviewed in 4, 5]. This study suggests that it extends even to the functional level, as evidenced, for example, by an increased abundance of BGCs encoding for RiPPs and NRPS in *L. sarmentosa* as compared to *Eunicella* specimens (see below).

**Supplementary Figures**


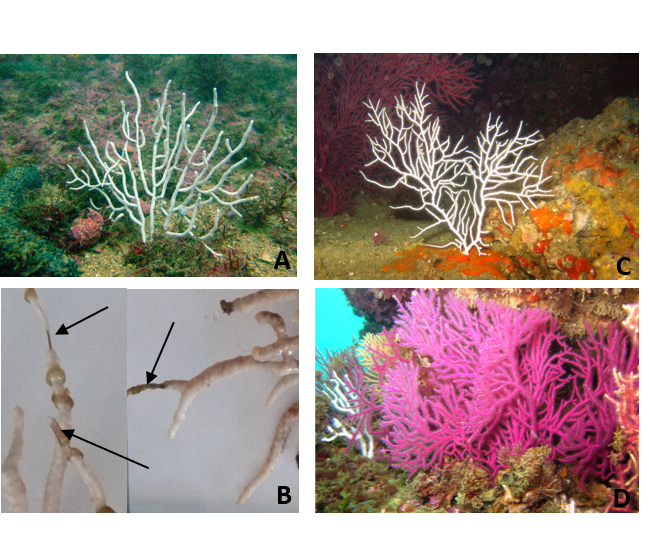


**Supplementary Figure S1.** Photographs of the octocorals investigated in this study. (**a)** healthy *Eunicella gazella,* (**b)** *Eunicella gazella* branches with necrosis signs (black arrows), (**c)** healthy *Eunicella verrucos*a, (**d)** healthy *Leptogorgia sarmentosa.* (**a**, **c** and **d**) are underwater photographs of the octocorals in their natural habitat taken off the Algarve coast (South Portugal).


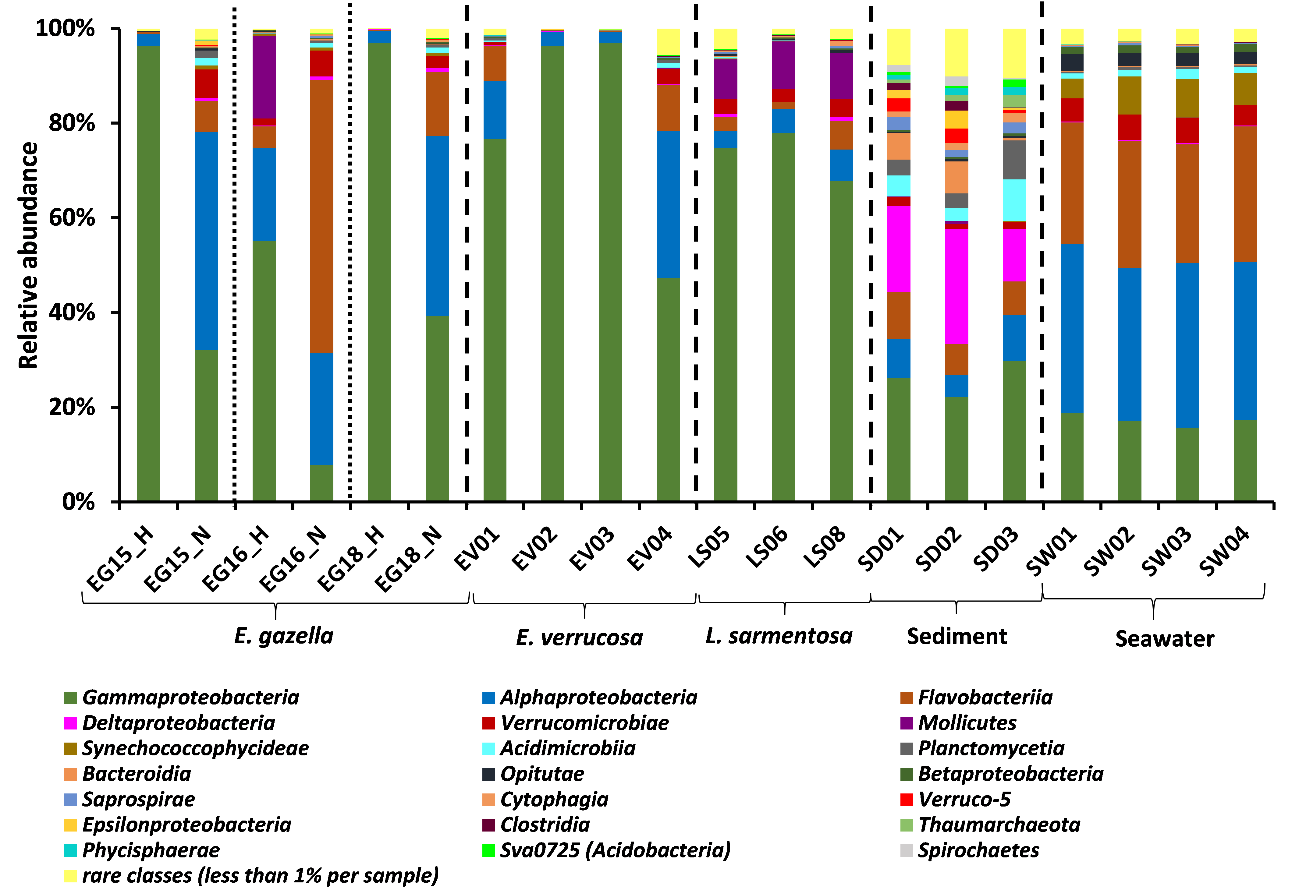


**Supplementary Figure S2.** Class-level prokaryotic community profiles of healthy (EG_H) and diseased (EG_N) *Eunicella gazella* tissue, healthy *Eunicella verrucosa* (EV01-EV04) and *Leptogorgia sarmentosa* (LS06-LS08) specimens as well as seawater (SW01-SW04) and sediment samples (SD01-SD03). Relative abundances are displayed for taxa representing more than 1% of the total dataset reads. Taxa with abundances below 1% across the data are collectively labelled as ‘rare classes’.


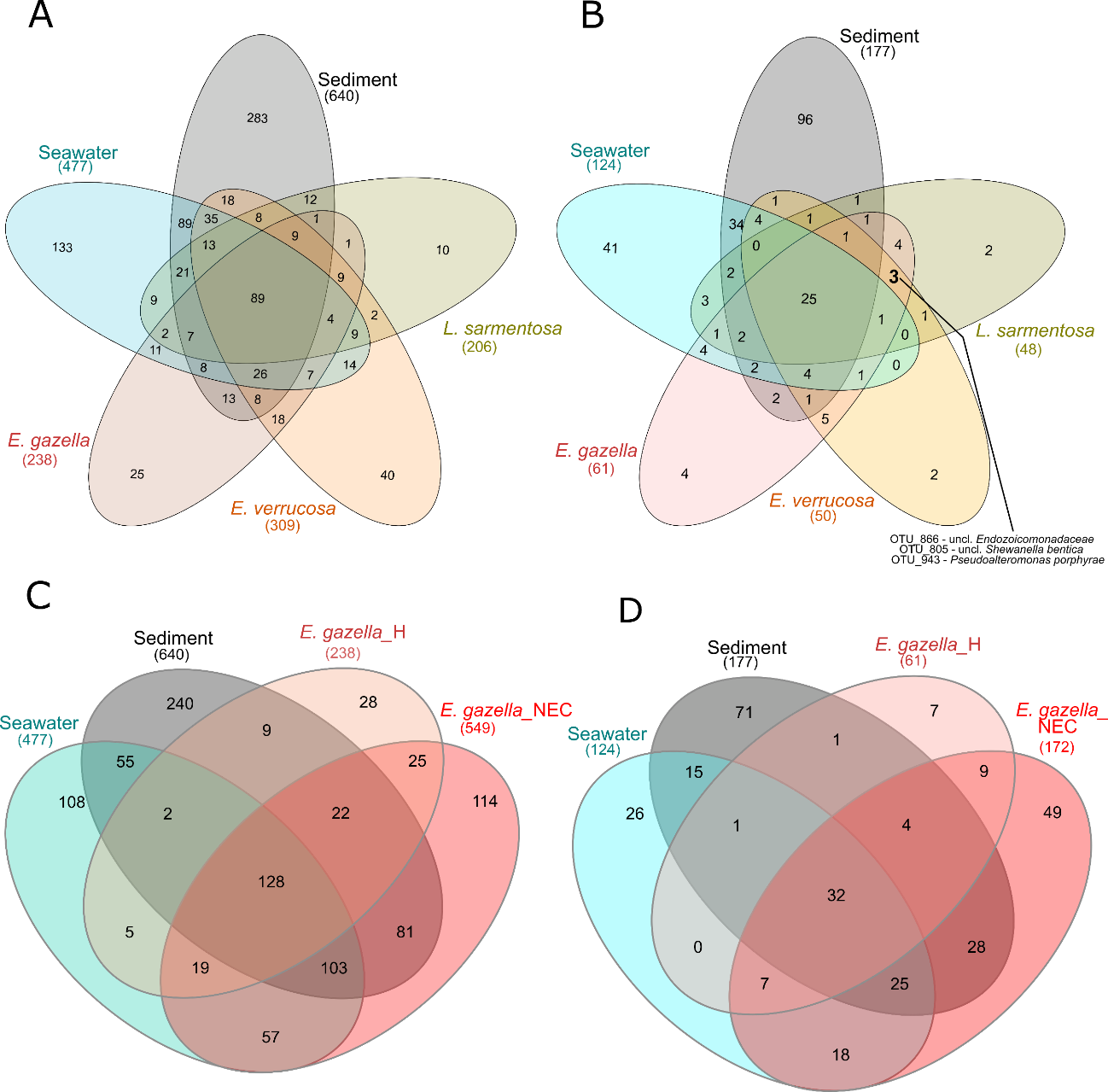


**Supplementary Figure S3.** Venn diagrams showing the shared and specific prokaryote phylotypes in octocorals and surrounding environments. (**a, b**) OTUs common and exclusive to seawater (blue), sediment (black) and healthy tissue of the octocorals *Leptogorgia sarmentosa* (olive), *Eunicella verrucosa* (orange) and *E. gazella* (salmon). (**c, d**) OTUs common and exclusive to seawater (blue), sediment (black) and healthy (salmon) *versus* diseased (red) tissue of *E. gazella*. (**a, c**) Replicate samples were pooled to portray the total number of prokaryote OTUs within each sample category. (**b, d**) Only those OTUs present in all replicate samples within each sample category are shown. The following three OTUs were shared only between all octocoral samples while not consistently detectable in sediment and seawater: OTU_866 *Endozoicomonadaceae*; OTU_805 *Shewanella bentica*; OTU_943 *Pseudoalteromonas porphyrae*. Venn diagrams were created using the online tool InteractiVenn (<http://www.interactivenn.net/> [6]).


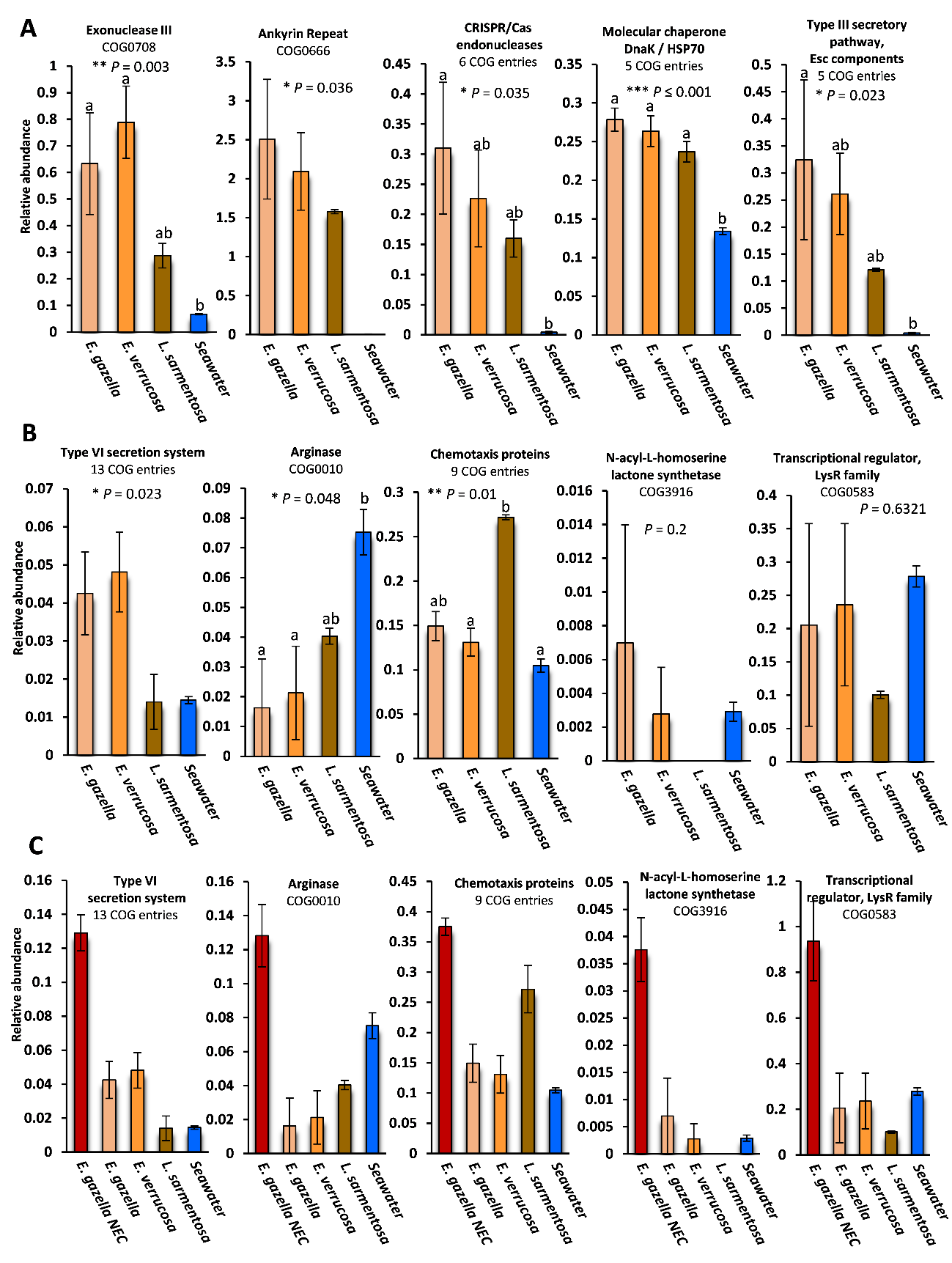


**Supplementary Figure S4.** Relative abundances of those Cluster of Orthologous Genes (COGs) of proteins in the microbiomes of the different octocoral species and seawater that were shown (in Figure 4) to be significantly enriched in the microbiomes of healthy (**A**) or diseased (**B** and **C**) *Eunicella gazella* tissue. Bars represent average proportions (%) ± standard errors. If a given function was represented by more than one COG entry across the dataset, the proportions of these functionally belonging COGs were summed and the number of COGs that contributed to each bar chart is given below chart titles; if only one COG entry contributed to a chart, the respective COG ID is given. If data presented normal distribution (Shapiro-Wilk test) and equal variance (Brown-Forsythe test), One-way-ANOVAs followed by Bonferroni *t*-tests for ‘all pairwise multiple comparison procedures’ were used to check for significant differences between sample groups. If one of the assumptions was violated, Kruskal-Wallis tests on ranks were instead performed, followed by Dunn’s post-hoc tests. Statistical significance was established at *P*-values ≤ 0.05. Letters above error bars indicate significant differences (* *P* ≤ 0.05; ** *P* ≤ 0.01; ****P* ≤ 0.001). Respective *P-*values are presented in the graphs. Panel (**c**) is equal to panel (**b**) but includes the necrotic *E. gazella* samples for an easier visualization of the “fate” of the respective functions in necrotic samples as compared to all healthy octocoral samples and seawater.

**References**

1. West PT, Probst AJ, Grigoriev IV, Thomas BC, Banfield JF: **Genome-reconstruction for eukaryotes from complex natural microbial communities**. *Genome Research* 2018, **28**(4):569-580.

2. van de Water J, Melkonian R, Voolstra CR, Junca H, Beraud E, Allemand D, Ferrier-Pages C: **Comparative assessment of Mediterranean gorgonian-associated microbial communities reveals conserved core and locally variant bacteria**. *Microbial Ecology* 2017, **73**(2):466-478.

3. van de Water JAJM, Voolstra CR, Rottier C, Cocito S, Peirano A, Allemand D, Ferrier-Pagès C: **Seasonal stability in the microbiomes of temperate gorgonians and the red coral *Corallium rubrum* across the Mediterranean Sea**. *Microbial Ecology* 2018, **75**(1):274-288.

4. van Oppen MJH, Blackall LL: **Coral microbiome dynamics, functions and design in a changing world**. *Nature Reviews Microbiology* 2019, **17**(9):557-567.

5. van de Water JAJM, Allemand D, Ferrier-Pagès C: **Host-microbe interactions in octocoral holobionts - recent advances and perspectives**. *Microbiome* 2018, **6**(64).

6. Heberle H, Meirelles GV, da Silva FR, Telles GP, Minghim R: **InteractiVenn: a web-based tool for the analysis of sets through Venn diagrams**. *BMC Bioinformatics* 2015, **16**(169).
